# Phylogenomic Insights into High Conservation and Lineage-Specific Expansion of the ABAPT Gene Family in Plants

**DOI:** 10.3390/ijms27083691

**Published:** 2026-04-21

**Authors:** Huan Song, Weiwei Li, Hong Xue, Mingming Zhang, Weiwei Zhang, Aoyu Chen, Lei Wang, Quanzhong Dong, Meng Zhang

**Affiliations:** 1 Soybean Research Institute, Keshan Branch of Heilongjiang Academy of Agricultural Sciences, Keshan, Qiqihar 161606, China; 2019songhuan@scbg.ac.cn (H.S.); liweiwei1618@163.com (W.L.);; 2 College of Agronomy, Northwest A&F University, Yangling 712100, China; 3 Department of Life Science and Agroforestry, Qiqihar University, Qiqihar 161606, China; zww121@163.com

**Keywords:** de-S-acylation, S-acylation cycle, phylogenomics, ABAPT, synteny relationships, lineage-specific expansion

## Abstract

De-S-acylation enzymes mediate the reversible S-acylation cycle and play critical roles in plant development and stress responses. However, the precise origin and evolutionary dynamics of this gene family in plants remain poorly understood. In this study, a total of 718 *ABAPT* genes were identified across 73 plant genomes, including 622 *ABHD17* and 96 *ABHD13* homologs, which share only a 20–30% conserved sequence identity between them. We further performed comprehensive analyses of gene duplication and structure, protein properties, synteny networks, and expression profiles to establish a systematic framework by classifying *ABAPT* genes in land plants. Our results revealed that *ABHD13* genes have been retained as a single copy in most angiosperm genomes, whereas *ABHD17* genes have undergone extensive expansion. *ABAPT* genes formed three major evolutionary clades: Clade 1 contained *ABHD13* homologs, while Clades 2 and 3 harbored *ABHD17* homologs. The three clades showed distinct disparities in intron–exon structural patterns and IDR properties. Phylogenomic synteny network analyses revealed the deeply conserved genomic syntenies within each of the six *ABAPT* subclades among the three clades, while Cluster4-Monocot was more dynamic and showed distinct lineage-specific duplication patterns restricted to Poaceae. *ABHD13s* exhibited constitutive expression patterns, while the tissue-specific expression genes were predominantly found within the *ABHD17s* subfamily. Notably, the *ABAPT8/9* subgroups were specifically expressed in reproductive organs, and the weighted gene co-expression network identified specific groups to find *ABAPT*-specific regulatory features, implying the presence of potential modules for the protein S-acylation cycle during pollen development. Additionally, our results suggested that C-terminal Cys-rich region was required for ABAPT10 localization. Altogether, this study sheds light on the evolutionary divergence of the *ABAPT* subclades across major green plant lineages and emphasizes the need for future functional characterizations.

## 1. Introduction

Protein acylation and de-acylation, the post-translational modifications (PTMs) of proteins, refer to the covalently selective attachment of acyl groups to peptide chains and their removal from proteins, respectively [1,2]. These two processes can enhance and reduce the liposolubility of proteins, correspondingly. With acyl-CoA often serving as the acyl donor, protein acylation can occur on different amino acids, including histidine, lysine, cysteine, glycine, and serine [3,4]. Distinct linkages and stabilities arise from the conjugation of acyl groups to diverse residues of amino acids with varying chain lengths, including myristoyl, palmitoyl, prenoyl (farnesyl), and acetyl moieties [2,5]. Long-chain palmitoylation typically forms a thioester bond (-S-CO-) with cysteine (S-acylation), which is chemically labile and enzymatically reversible, making these processes even more attractive for investigations [6]. Lipid acylation confers hydrophobicity to target proteins; however, accumulating evidence highlights that the biological implications of S-acylation and the de-S-acylation enzyme can act more than membrane anchoring [1]. Notably, even membrane proteins inherently harboring a transmembrane domain (TMD), such as CESA6, can be S-acylated [7]. The S-acylation may determine a multitude of properties and behaviors of cellular proteins, such as vesicle trafficking, stress responses, phosphorylation and ubiquitination, and protein–protein interactions (PPIs), thereby influencing vital cellular processes including cell growth, substance transport, and signal transduction [1,8,9,10].

Protein S-acylation is primarily catalyzed by protein acyltransferases (PATs), whereas de-S-acylation is predominantly mediated by acyl-protein thioesterases (APTs) [4]. It is estimated that around 6% of detectable proteins in Arabidopsis proteome are modified by S-acylation. On the other hand, only a limited number of PATs and APTs implicated in this process have been documented in cells, implying that this process is ubiquitously conserved and may exhibit limited substrate specificity [9]. S-acylated proteins in cells also exhibit pronounced tissue specificity with unique or non-overlapping acylomes across different plant tissues. For example, only 71 consistently identified proteins were confirmed to be S-acylated among 1094 peptides across all six tissues under high- and medium-confidence thresholds, whereas 516 proteins were uniquely detected in only one tissue retrieved from a tissue-specific acylome [9]. Therefore, it implies that S-acylation is an important PTM involved in plant growth and development.

Although extensive insights have been gained into the roles of S-acylated proteins and PATs, the complete S-acylation cycle between palmitoylation and depalmitoylation has rarely been investigated in plant cells, even though not all S-acylated proteins undergo de-S-acylation [10]. There is a persistent gap in our knowledge of de-S-acylation enzymes, especially for green plants. The recent description and characterization of eukaryotic depalmitoylation enzymes—including human Alpha/Beta-Hydrolase Domain17 and 13 (ABHD17 and ABHD13), APT1/2 (APT1:LYPLA1, APT2:LYPLA2), and MtAPT1 from *Medicago truncatula*—highlight the necessity of further research in this field [3,11,12]. De-S-acylation proteins involved in neuronal synaptic activity (e.g., PSD-95) and cancer growth (e.g., N-Ras) have recently garnered attention, and these studies have extended our understanding of palmitoylation and depalmitoylation in humans and other animals [3,13,14]. It has also been reported that loss-of-function mutations in ABHD13 homologs of fungi *Aspergillus fumigatus* and *Neurospora crassa* result in reduced spore germination, but none of these mutants showed lethality or significant morphological alterations. Moreover, the ABHD13 protein in *A. fumigatus* has been linked to polarized hyphal growth [15,16]. However, little progress had been made in identifying protein de-S-acylation enzymes and their substrates in plants until the development of an ABHD17-like hydrolase co-transformed screening system in 2021 [6]. This path-breaking research paved the way for the identification of responsible ABAPTs (alpha/beta-hydrolase domain-containing protein 17-like acyl protein thioesterases) from Arabidopsis that catalyze the de-S-acylation of several key components involved in pathogen resistance [6,17]. Recently, BR signaling components, BSKs, are primarily associated with membranes via S-acylation. Salicylic acid (SA) determined the plasma membrane targeting of BSK1 via de-S-acylation mediated by ABAPT11, leading to the disruption of its localization and function with regard to BR-mediated plant growth [18]. These studies have revealed the significance of protein de-S-acylation in plant life activities. However, the ubiquity of this process contrasts sharply with the scarcity of existing reports. The complexity of ABAPT family members requires an overall understanding of their evolution and structure. Unfortunately, the evolutionary trajectory and functions of the ABAPT homologous member remain unclear in many plants, preventing it from keeping pace with fields of PTMs.

The ABAPT family described in ESTHER (https://bioweb.supagro.inrae.fr/ESTHER/, accessed on 21 February 2025) has been categorized into the alpha/beta-hydrolase fold (ABHD) superfamily, with the alpha/beta-hydrolase fold domain as a defining characteristic [19,20]. The ABHD superfamily comprises widespread and functionally malleable proteins whose core fold is highly conserved and consists of a five to eight-stranded β-sheet (parallel or mixed) surrounded by α-helices [20]. The past two decades have witnessed the unraveling of this superfamily in terms of structural and functional variation. Nowadays, more than 200,000 various ABHDs have been included in the ESTHER database by 2023. Specifically, ABAPTs also belong to the Hydrolase_4 superfamily in block X, which also includes monoglyceride lipase/lysophospholipase (MAGL) and human ABHD12. ABAPTs can be further subdivided into ABHD17-depalmitoylase and ABHD13-bud emergence 46 (BEM46). These two proteins often share a surprisingly low sequence identity while maintaining a highly conserved three-dimensional core architecture. Hence, the similar function and topology of the ABAPT subfamily imply that they may share a common ancestor in green plants. However, due to sequence redundancy, domain similarity, and the extensive expansion or contraction of ancestral genes, phylogenetic reconstruction alone sometimes fails to clearly resolve the evolutionary history of these genes [21,22,23]. Considering the advantages of syntenic analysis and network-based analysis [23,24,25], it is imperative to apply these approaches to the analysis of the plant *ABAPT* gene family, thereby deciphering their evolutionary relationships.

In this study, we first inferred the phylogeny for *ABAPT* homologs recovered from extant green plant lineages ranging from charophytes, liverworts, mosses, lycophytes, and ferns to angiosperms and analyzed gene duplication events within the *ABAPT* family. Subsequently, we exploited a pipeline incorporating phylogenomic and syntenic methods to provide an initial framework for further functional studies of plant *ABAPT*s. Finally, the expression pattern of *ABAPTs* from representative species was investigated, and a pollen development WGCNA network was constructed as an example for unveiling the potential functions of the de-S-acylation enzyme in a model plant. This study may facilitate a comprehensive understanding of dynamics cycling between palmitoylation and depalmitoylation in plants, as well as highlight the importance of further work in this field.

## 2. Results

### 2.1. Identification of de-S-Acylation Proteins in Plants

**Figure 1:**
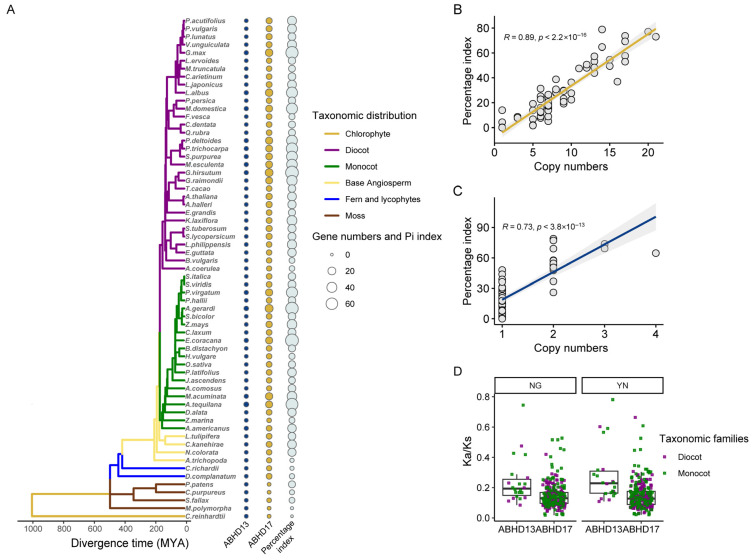
Distribution, classifications, and selection pressure of *ABAPTs* in plants. (**A**) Species tree with the corresponding gene copy number of *ABHD13/17* subfamily in each species was generated using the Timetree5 database. The percentage index denotes the ratio of numbers of collinear gene pairs to the total numbers of genes. Evolutionary information and percentage index for additional species are not collected by Timetree5 but provided in Table S1. (**B**,**C**) Correlation between the copy numbers of *ABHD17s* (**B**) or *ABHD13s* (**C**) and the percentage index (Pi index) across different investigated species. *p* value was calculated from Pearson’s correlation test, and the trend line was drawn using the LOWESS (locally weighted scatterplot smoothing) method. Correlations were significant at *p* < 0.05 for all subfamilies. (**D**) The ratio ranges of Ka/Ks of collinear *ABAPT* gene pairs derived from whole-genome duplication in angiosperms, calculated using the NG (left) and YN (right) methods.

To identify de-S-acylation enzymes in plants, we collected a total of 11 ABAPT members (ABAPT1–ABAPT11) from Arabidopsis, which have been recently characterized as a de-S-acylase family in plants [6]. Full amino acid sequences of putative ABAPT family members were then deduced using the Arabidopsis de-S-acylation enzymes as queries by local BLASTp tools. We also performed a hidden Markov model (HMM) profile search using Hydrolase_4 (PF12146) for potential ABAPT proteins in 73 fully assembled green plant genomes retrieved from Phytozome V13 and Ensembl Plants database (Table S1). Combining two different approaches, a total of 740 ABAPT homologs were initially identified across the surveyed plant species (Table S1). However, some of the putative ABAPTs were excluded from a preliminary phylogenetic analysis whose homology could not be confidently assigned due to sequence redundancy within the superfamily (see Table S1). These sequences mainly belonged to the MAGL subfamily arranged in the Hydrolase_4 superfamily. Therefore, sequence identification of HMM-based methods was improved using ABHD13- and ABHD17-specific HMM profiles obtained from the ESTER database. A total of 718 ABAPT proteins were retrieved from 73 plant genomes, including 622 ABHD17s and 96 ABHD13s after filtering out sequences with an incorrect start codon or those encoding fewer than 150 amino acid residues (Table S1). These 718 protein sequences were then subjected to the InterProscan and Pfam database to confirm the presence of the conserved Hydrolase_4 domain. We observed that both *ABHD13s* and *ABHD17s* are ubiquitously distributed across all major plant lineages including charophytes, hornworts, liverworts, mosses, lycophytes, ferns, and angiosperms, suggesting an origin earlier than that of chlorophytes for *ABAPT* (Figure 1A). Moreover, multiple copies of *ABHD17* were observed in all land plants, and the abundance of *ABHD17* in plants significantly surpasses that of *ABHD13* (Figure 1A; Table S1). The copy numbers of *ABAPT* were lower in the basal lineages of green plants like algae and exhibited greater variation in both sequence length and similarity compared with other land plants. For instance, ABAPT members from chlorophytes shared only 30–40% sequence identity with those from land plants such as Arabidopsis. In contrast, ABAPTs from land plants (mosses, lycophytes, ferns, and angiosperms) showed a 60–99% sequence identity at the protein level, indicating that the protein sequences of these archetypal ABAPT homologs are highly conserved from mosses to angiosperms (Figure S1). Notably, the two ABAPT subfamilies, ABHD13 and ABHD17, shared low sequence conservation (20–30% identical amino acids; Figure S1). Sequence similarity within the ABAPT family was also significantly higher than that of PATs (DHHC-domain PATs), reflecting the high evolutionary conservation of ABAPTs (Figure S1). Overall, *ABHD13* existed as a single copy in most species, with a maximum of no more than four. In contrast, *ABHD17* occurs as multiple copies in some lower mosses (e.g., *Sphagnum*) and all higher plants, ranging from five members in *Amborella trichopoda* to 21 in *Andropogon gerardi* (Figure 1A). These results indicated that the *ABAPT* gene family has expanded mainly via the *ABHD17* subfamily during plant evolution.

### 2.2. Lineage-Specific Expansions Shaped the Diversity of the ABAPTs in Plants

Polyploidization is a crucial determinant of adaptive evolution [26], and we hypothesized that the expansion of *ABAPTs* in land plants may have been shaped by this process. We therefore identified gene duplication events, and our results showed that WGD or segmental duplication mainly contributed to the *ABAPT* expansion, with 347 genes assigned to this duplication mode across 69 plant species (Table S2). A total of 118 and 122 duplicated gene pairs were classified as transposed or dispersed duplication events, respectively. However, other gene duplication types—such as tandem and proximal duplication—played a minor role, accounting for less than 2% of all homologous genes (Table S2). Meanwhile, WGDs or segmental duplications represented a uniquely important driving force underlying the expansion of *ABHD13* genes in dicots such as soybean (*Glycine max*), cabbage (*Brassica oleracea* var. *capitata*), and turnip (*Brassica rapa* var. *rapa*), as well as in monocots including wheat (*Triticum aestivum*) and switchgrass (*Panicum virgatum*) (Figure 1C; Table S2). In most angiosperms, *ABHD13* copies were generally localized in similar genomic regions and shared analogous flanking gene contexts, but its syntelogs were found to be absent from its conserved syntenic genomic regions (Figure S2). *ABHD13* genes have largely restored single-copy status in most angiosperms following multiple rounds of ancestral plant paleopolyploidization (Figure S2). As supporting evidence, 213 collinear orthologs were identified between the genomic collinear region in Arabidopsis (Chr5–Chr3, with Ks_mean = 1.31, corresponding to beta events), which retained only one parental *ABHD13* copy in chromosome 5 (Figure S2). Similarly, in the monocot sorghum, *ABHD13* genes located in the conserved syntenic region (293 collinear orthologs with Ks_mean = 2.31) were lost in another genomic duplication block (Chr2–Chr1, lost in Chr1), which is congruent to the rho event (Figure S2). The genome of *Vitis vinifera* (wine grape) has been shaped only by the ancient γWGT event and is widely used as a reference for ancestral karyotype inference [23]. Intra-species synteny analysis indicated that *ABHD13* underwent a potentially triplicated duplication corresponding to triplicated collinear blocks in the grape genome, and only one copy of *ABHD13* (*VIT_211s0016g01490*, Chr11) was retained after the gamma event (Figure S3). We found that *ABHD13* genes were preferentially lost compared with their flanking genes, probably via gene-specific deletion rather than the removal of large genomic blocks—a pattern consistent with ancestral polyploidization events (evidenced by higher Ks values across all species) (Figure S2). Thus, we deduced that the *ABHD13* counterpart may have been deleted to revert to a singleton status following multiple whole-genome duplications/triplication events (e.g., ρ WGD and γ WGT in the Poaceae family). However, in some species like soybean, which its genome underwent two rounds of WGD events around 58 and 13 million years ago [27], the most recent WGD events (Ks_mean = 0.08) contributed to the generation of two nearly identical ABHD13 copies. Collectively, WGD, followed by TRD and DSD, has driven the evolution of the ABAPT gene family, which also reflected in linear regression relationship between the copy numbers and percentage index of gene polyploidization (Figure 1B,C).

**Figure 2:**
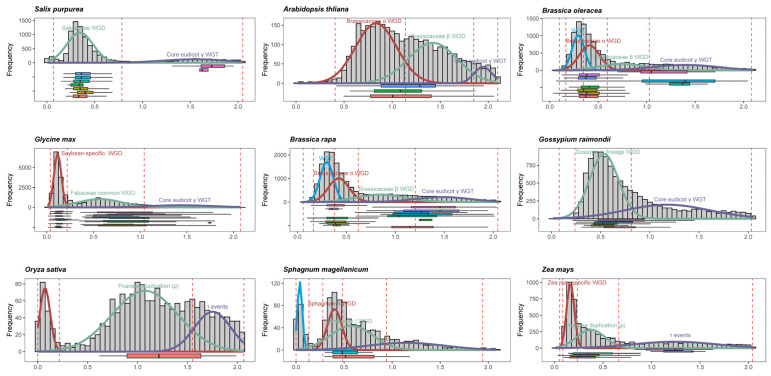
Ks distributions for multiple polyploidy events in different plant lineages. Distributions of synonymous substitution rates (Ks) of the *ABAPT*-anchored syntenic blocks and total paralogous gene pairs retained on syntenic blocks in representative species. Boxplots depict the Ks distributions of syntenic blocks containing *ABAPT* family members, and corresponding polyploidy events in different lineages are marked according to previous reports, including Poaceae rho WGD [28], Fabaceae common WGD [27], Salicaceae WGD [26], Brassicaceae alpha/beta WGD [26], core eudicot gamma WGT [29], and Sphagnum common WGD [30]. WGD-derived gene pairs with Ks ≥ 2.0 were excluded due to substitution saturation at high Ks values. Red lines represent the mean ± N standard deviations of the corresponding peaks (default = 2), and the distributions of substitution rates of corresponding peaks were visualized using differently colored normal curves.

Since homologous gene loci within colinear blocks are considered as the products of lineage-specific WGD events, we conducted an in-depth analysis of lineage-specific and species-specific *ABAPT* expansion integrated with Ks distribution patterns, aiming to elucidate the consequences of gene duplication (Figure 2). As expected, lineage-specific duplication events greatly influenced the diversification of the *ABAPT* gene family. For instance, the most recent WGD-derived gene pairs were characterized by an early Salicaceae-specific polyploidization that yielded seven *ABAPT*-containing blocks in *Salix purpurea* (Figure 2). In soybean, novel *ABAPT* paralogs have emerged as the result of two WGD events in ancestral legume species (nine blocks) and the *Glycine* genus (eight blocks), respectively. The large expansion of *ABAPTs* in *Brassica rapa* and *Brassica oleracea* was consistent with their recent WGT compared with Arabidopsis, which shared an ancestral α/β WGD event in the Brassicaceae family (Figure 2). Notably, the earliest multiple copies of ancestral *ABAPT* can be monitored in bryophytes. Three moss *ABHD17* homologs were found to be located in syntenic blocks in *Sphagnum fallax* and *Sphagnum magellanicum*, which may be attributed to the WGD events shared by *S. angustifolium* and *S. divinum* (Figure 2), with two Ks peaks (0.58 and 0.41) dated to approximately 189–247 Ma and 102–122 Ma based on mixture models. The Ka/Ks ratio for all WGD-derived gene pairs indicated that *ABAPT* genes were highly conserved and had experienced a purifying selection in land plants, including mosses and lycophytes (Figure 1D; Table S3). Moreover, the Ka/Ks ratio of *ABHD13s* was relatively higher than that of *ABHD17s*, and elevated Ka/Ks ratios of *ABAPT* genes were more common in monocots than in eudicots (Figure 1D; Table S3).

### 2.3. Domain Structure and Motif Analysis of ABAPTs in Representative Species

**Figure 3:**
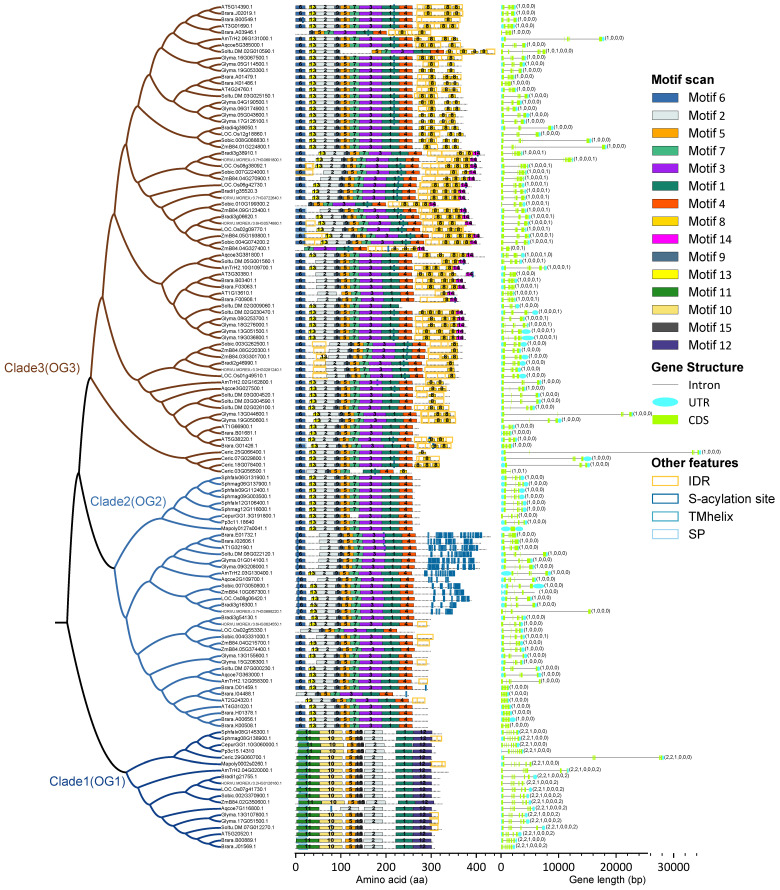
Phylogenetic and motif analyses of ABAPTs. The unrooted majority-rule consensus phylogenetic tree, conserved motif, and gene structures were constructed using MrBayes 3.7 program and MEME software. Different motifs are represented by different colors, and gene structures were annotated according to gff3 files from merged genome annotations of multiple species. Conserved domains or motifs are displayed using color-coded boxes and defined as follows: IDR, consensus disorder region; SP, signal peptide region; TMhelix, transmembrane domain; and S-acylation site, S-acylated residues predicted using CSS-Palm 4.0. Orthologous groups on tree branches are indicated with different colors.

Since no phylogenetic study of de-S-acylation proteins has been reported, we selected a subset of species from 17 species representing five mosses, one fern, and 11 land lineages (one basal angiosperm, five eudicots, and five monocots) to construct preliminary phylogenetic trees using Bayesian inference and maximum-likelihood analysis, along with a survey of motifs and gene structures, to further clarify the topological relationships among ABAPT family members (Figure 3 and Figure S4). Three distinct clades were identified with almost all branch bootstraps being well supported (posterior probability = 1), which is consistent with phylogenetic orthology (OG) inference using OrthoFinder2 (Figure 3 and Figure S4; Table S4). Clade 3 (OG3) accounts for more than half of all ABAPT members, far exceeding its sister branch Clade 2. Additionally, the number of amino acid residues in Clade 3 members is generally higher than that of most proteins from other clades (Figure 3). All *ABHD13* members of Clade 1 formed a monophyletic cluster, and their sequences exhibited considerable differences in the intron number compared with other clades (e.g., Clade 2). Despite the high overall conservation of *ABHD13* genes, angiosperm *ABHD13s* harbor seven introns with a phase pattern of 2, 2, 1, 0, 0, 0, 2, whereas those from lower plants contain merely six introns with a phase pattern of 2, 2, 1, 0, 0, 0. This indicates that an intron gain event occurred at the C-terminus of ABHD13 in angiosperms (Figure 3). The intron phase had already been established and subsequently fixed in bryophytes and remained evolutionarily conserved in the lineage extending to angiosperms. Phylogenetic analysis revealed that Clade 2 of OG2 was represented by both mosses and angiosperms and exhibited identical gene structures. They contained four introns with the phase pattern of 1, 0, 0, 0 in their respective gene annotation files, with the exception of *Marchantia polymorpha*. The sister branch of the moss cluster (*ABAPT10* subclade in Clade 2) displayed a notably longer nucleotide length of open reading frame (ORF) than those of other groups (Figure 3). Within Clade 3, the four members from *Ceratopteris richardii* formed a sister branch to other *ABHD17*s and contained 4–6 introns in angiosperms with phase profiles of 1, 0, 0, 0; 1, 0, 0, 0, 1; and 1, 0, 0, 0, 1, 0. These features suggested that an intron gain event (involving 1–2 introns) likely occurred at the C-terminus in angiosperms relative to their ancestral species. In some ABHD17 and ABHD13 members, abundant IDRs were identified in their C-terminal domains, especially in the Clade 3 members with relatively long IDRs (length ≥ 50 amino acids) (Figure 3). IDRs play multifunctional roles in plant adaptation to novel environments, such as serving as interaction sites for recognizing multiple partners [31]. In addition to Clade 3, IDRs are occasionally found within ABHD13 and Clade 2, but these proteins exhibit a relatively higher similar and well-organized C-terminal composition compared with Clade 3 (Figure 3). Moreover, Clade 3 harbors a unique Motif 8 that only presents at the C-terminal region in a tandem arrangement, wrapping around the IDR location (Figure 3).

### 2.4. Subcellular Localization of AtABAPT2 and AtABAPT10

In animals, N-terminal cysteine clusters (5Cys) are essential for ABHD17A auto-palmitoylation and act as crucial determinants for its de-S-acylation activity [3,13,14]. Therefore, we predicted the palmitoylation sites of ABAPT members, and the results showed that most of the S-acylation sites are localized within Motifs 1 and 3 (Figure 3). All angiosperm members belonging to the ABAPT10 (AT1G32190) subclade in Clade 2 exhibited a dense cluster of Cys residues at their C-terminus, reminiscent of a well-studied multiple S-acylated proteins CESA—a family essential for cellulose synthesis [9].

**Figure 4:**
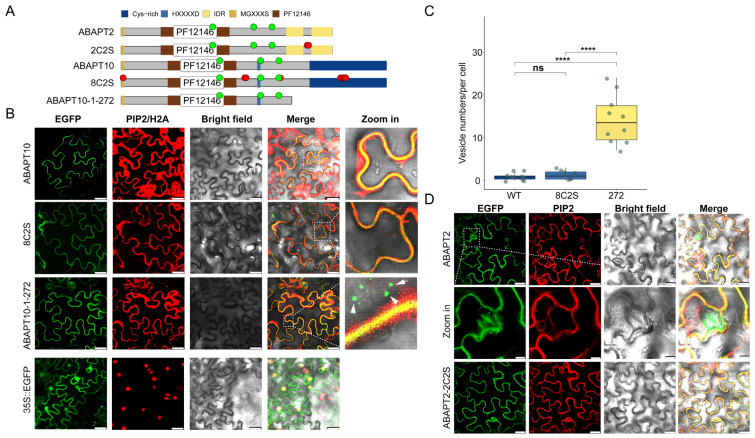
Subcellular localization of AtABAPT2 and AtABAPT10. (**A**) Schematic representation of full-length ABAPT2/10, C-terminal truncated mutant ABAPT10-1-272, and their mutated S-acylation site variants. The conserved catalytic residues (Ser, His, and Asp) are marked with green dots, whereas the predicted S-acylated Cys residues are highlighted in red dots. 2C2S represents a mutation of ABAPT2^C295/296S^, and 8C2S represents a mutation of ABAPT10^C3/196/197/251/348/352/353/354S^. (**B**) Fluorescent imaging of 35S::ABAPT2-eGFP, 35S::ABAPT10-eGFP, 35S::ABAPT2-2C2S-eGFP, 35S::ABAPT10-8C2S-eGFP, 35S::ABAPT10-1-272-eGFP, and 35S::eGFP with the plasma membrane marker AtPIP2-mCherry or nuclear marker H2A-mCherry transiently expressed in tobacco leaves. Scale white bars: 25 μm. White arrowheads indicate vesicle-like structures. Control, showing that the GFP signal from the 35S::GFP construct, was distributed throughout the cell. (**C**) Quantification of vesicle number per cell was performed using ImageJ V2-based counting. Asterisks indicate statistically significant differences (****: *p* ≤ 0.0001, Student’s *t*-test, n = 10, and ns: no significance). (**D**) Fluorescent imaging of 35S::ABAPT2-eGFP and 35S::ABAPT2-2C2S-eGFP with the PM marker AtPIP2-mCherry transiently expressed in tobacco leaves. Scale bars: 25 μm. Zoom in: 8 μm.

Previous reports indicated that AtABAPT2 and AtABAPT10 contain multiple S-acylated cysteines, which were experimentally confirmed by the acyl-resin-assisted capture (acyl-RAC) assay [9]. Here, their subcellular localization was further determined by being transiently expressed in *N.benthamiana* leaf epidermal cells. Results showed that ABAPT2/10 localizes to the plasma membrane, as indicated by the co-localization with the PM marker protein AtPIP2-mCherry (Figure 4). Additionally, the fluorescence of ABAPT2-eGFP was also observed in the nucleus. Notably, a strong signal of eGFP was observed in stomatal cells (Figure 4D). To further determine the effect of S-acylation of ABAPT2/10 on their PM location, we performed site-directed mutagenesis to generate ABAPT2/10 mutant variants in which all S-acylated cysteine residues were replaced with serine (Figure 4A). In addition, cysteine-rich (C-rich) region (ABAPT10-1-272) deletion constructs were created to investigate whether these cysteine-rich clusters are essential or sufficient for membrane localization (Figure 4A). Unexpectedly, we found that these cysteine-replacement mutants were still capable of anchoring ABAPT2/10 proteins to the PM, suggesting that S-acylation is not the sole key determinant for the localization of ABAPT2/10 to PM (Figure 4B,D). However, some additional fluorescence was consistently observed in vesicle-like clustering structures beyond the PM in cysteine-rich region truncated mutants (ABAPT10-1-272), which were clearly separated from the PM marker (Figure 4B). Such structures were nearly absent in the wild-type (WT) and 8C2S variants (Figure 4C). These smaller vesicles may represent endosomal compartments, and fluorescence was consistently observed after different incubation periods (Figure S5). This result suggests that such a peptide fragment is crucial for the ER-to-PM trafficking of newly synthesized ABAPT10 [11,32].

Overall, all the ABAPT members shared conserved catalytic residues (Ser, Asp, and His) in the ABHD region with high sequence identity, while C-terminal sequences varied among different subclades. ABAPT members can be further divided into three subtypes: Clade1 of ABHD13, Clade3, characterized by the common long-IDR feature, and the occurrence of Cys-rich clusters in Clade 2 genes (Figure 3). Additionally, our results showed that moss-derived ABHD17 sequences clustered together with Clade 2 members, suggesting a possible early derivation of ABHD17 from this clade. Gene structures varied considerably among different clades but were relatively conserved within each clade. Therefore, further evidence is needed to support and refine the classification of ABAPTs.

### 2.5. Phylogenomic Synteny Network Analyses of ABAPT Genes

**Figure 5:**
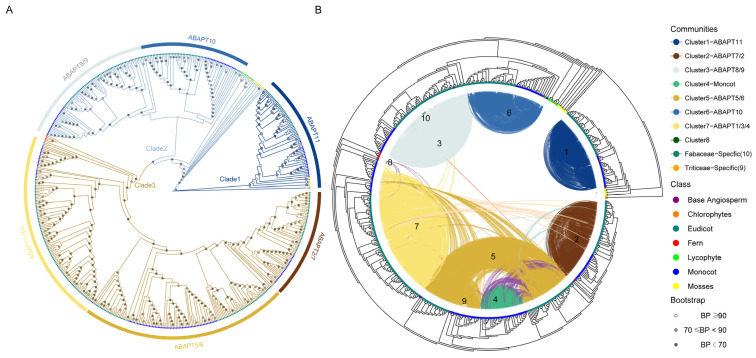
Maximum-likelihood gene tree of the ABAPT gene family and their syntenic relationships. (**A**) Phylogenetic analysis of the ABAPT members in 59 representative green plants. Branch circles on the nodes represent ultra-bootstrap values from 10,000 bootstrap replicates (90 or greater, white; 70 to 90, gray 60; while below 70, gray 40), and organisms are categorized into their floristic system based on Timetree5 taxonomic classification. Leaf nodes are colored to distinguish the classes of green algae, mosses, lycophytes, ferns, basal angiosperms, eudicots, and monocots, respectively. The outer rings represent ABAPT subclades according to their phylogenetic relationship. (**B**) Syntenic relationships are illustrated within each community. The numbers annotated on the tree denote the community IDs in Figure S10. Each connecting line, which is located inside the inverted circular gene tree, indicates a syntenic relationship between two ABAPTs. The connecting lines are colored according to the identified communities in Figure S6. Small-scale communities (Clusters 4, 8, 9, and 10) belonging to primary subclades (ABAPT5/6, ABAPT1/3/4, ABAPT5/6, and ABAPT8/9) are categorized into lineage-specific synteny communities, which are derived from transpositions and/or fractionation after WGD.

Synteny information may contribute to elucidating the shared ancestry of genes and resolving ambiguous gene orthology relationships in cases where phylogenetic methods may be inconclusive owing to paralog redundancy in multigene families [21,33]. Phylogenetic and synteny network analyses were then performed to reconstruct the orthogroups in 59 green plants, including three charophytes, four mosses, one lycophyte (*Diphasiastrum complanatum*), one fern (*Ceratopteris richardii*), and 50 angiosperms (Tables S1, S5 and S6; Figures S6 and S7). Considering the large evolutionary distance between the 50 angiosperms and the other nine non-angiosperm species, we separately applied the Syntenet pipeline for syntenic block identification, network construction, and community detection [25]. Community clustering analysis revealed that the *ABAPT* family can be further classified into six main subclades—consistent with our preliminary phylogenetic trees inferred using Arabidopsis genes as references (Figure 3 and Figure 5). Clade 1, corresponding to *ABHD13* (*ABAPT11*), encompass a distinct angiosperm-wide synteny community with no syntelogs linked to other clades. This suggests that this subfamily split apart from a more ancestral organism than mosses, probably occurring in algae or earlier eukaryotic lineages (Figure 5, Figures S6 and S7). Clade 2 comprises two subclades corresponding to *ABAPT10* and *ABAPT8/9*. The former is independent of other syntenic clusters, whereas *ABAPT8/9*s are weakly associated with Clade 3 (Figure 5, Figures S6, S8 and S9). This indicates that a common ancestor before angiosperm radiation may have undergone duplication to give rise to *ABAPT10* and *ABAPT8/9*, followed by further diversification after the divergence of mosses (Figure 3 and Figure 5). Moreover, three subclades within Clade 3 displayed extremely strong syntenic conservation among their orthologs, including *ABAPT1/3/4*, *ABAPT5/6*, and *ABAPT7/2,* with weak syntenic connections among these three subclades (Figure 5). The lineage-specific syntenic Cluster 4 (*ABAPT5/6* within monocots) is confined to Poaceae (Figure 5 and Figure S10). These synteny results were highly consistent with the tree topology inferred from phylogeny reconstruction. Additionally, the inclusion of four *Ceratodon purpureus ABHD17* homologs in Clade 3 suggests that the origin of Clade 3 genes probably traces back to fern radiation (Figure 5). Since the quality of the phylogeny results is expected to be directly affected by gene duplication and loss as well as incomplete lineage sorting, we further investigated the topological reliability of gene pairs of syntelogs between Clade 2 and Clade 3. Indeed, the discordance between the syntelogs and HMMERlogs (i.e., defined as homologs with an identical domain composition) was consistent with the maternal tree (Figure 5B, Figures S8 and S9). Notably, *ABAPT* loci were covered by colinear blocks in lycophytes (*Diphasiastrum complanatum*) and mosses. For example, two *ABHD17* homologs share obvious gene collinearity between *P. patens* and *C. purpureus*, reflecting the highly conserved genome content in extant representative mosses (Figure S7). Analysis of angiosperm-wide synteny revealed that, during the evolution of angiosperms, even when the differentiation between monocots and dicots occurred, the ancestral gene loci was still retained—including communities 1, 2, 3, 5, 6, and 7 (Figure S10). Lineage-specific synteny is often more dynamic with respect to genomic position—for instance, community 4 is Poaceae-specific, whereas community 9 comprises *Triticum* and *Hordeum* species (*Triticum aestivum* and *Hordeum vulgare*), highlighting the dynamics of *ABAPT* genomic contexts in certain lineages (Figures S6 and S10). These results suggested that the archetype *ABAPT* loci was extremely conserved in terms of syntenic organization across both land plants and lower plants, with infrequent changes in their genomic contexts. Collectively, multiple duplication events occurred prior to angiosperm origin and have been retained as angiosperm-wide synteny ever since, giving rise to three main *ABAPT* clades constituted by six subclades.

### 2.6. Poaceae-Specific WGD Duplication Events and Gene Loss After Duplication in ABAPT5/6

**Figure 6:**
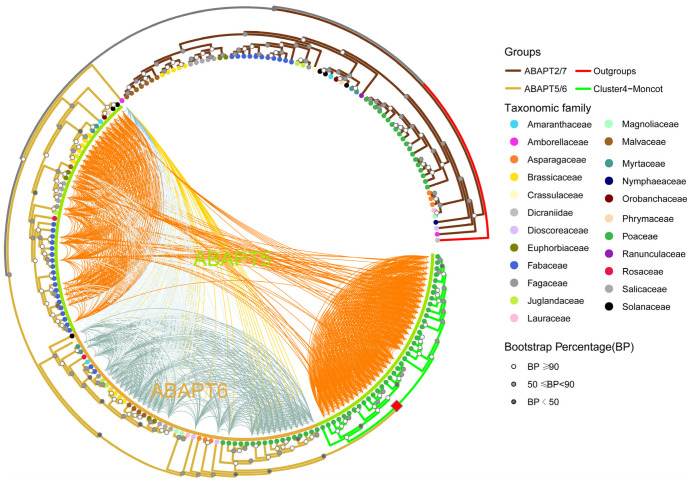
Maximum likelihood (ML) tree of ABAPT5/6 in Clade 3. ABHD17 (*CepurGG1.3G191800*) from *Ceratodon purpureus* is used as an outgroup. Putative WGD events (with Ks values around 0.68 in most Poaceae species) that occurred in Poaceae are indicated by red diamond (Table S3). Branches are decorated consistently with those in Figure S6. Colored lines indicate strong conservation of synteny between gene pairs within and between ABAPT5/6 subgroups. The ABAPT2/7 subclade was retained here without synteny relationships to better represent the orthologous synteny assignments in the ABAPT5/6 subclade. The phylogenetic tree was inferred by ML analysis using 1000 bootstraps.

Independent and lineage-specific clusters arise primarily from transpositions and/or extreme gene fractionation after WGD [23,24]. For the *ABAPT5/6* subclade, widespread syntelogs were detected between dicot genes and the Poaceae-specific Cluster 4, indicating that the formation of such lineage-specific clusters resulted from gene loss rather than transposition events (Figure 5 and Figure S6). Integration of intra-species duplication and inter-species synteny analyses revealed that numerous duplicated genes were retained from the shared WGD event, with conspicuous Ks values ranging from 0.61 to 1.10. Their occurrence is consistent with the divergence level of paralogues derived from core grasses (Figure 6; Table S3). For the *ABAPT5/6* subclade in monocots, the members of Cluster 4 were syntenically unrelated to another monocot group within the *ABAPT5/6* subclade. Additionally, the syntenic affinity of Cluster4-Monocot was closer to the *ABAPT5* (AT3G30380 located branch) of dicots than the *ABAPT6* (AT3G30380 located branch). By comparison, the other monocot group (sister to Cluster4-Monocot) maintains predominant synteny conservation specifically with the *ABAPT6* (Figure 6 and Figure S6). Given that syntenically conserved gene pairs usually represent homologous genes descended from a common ancestor, and orthologous synteny is highly informative for reconstructing evolutionary histories [34], we postulated that the Cluster4-Monocot and the *ABAPT5* of dicots originated from a common ancestral gene, whereas the other monocot group shared a common ancestral origin with the *ABAPT6*. Moreover, ancient Poaceae-specific WGD duplications occurred after the divergence of monocots, giving rise to the large Cluster4-Monocot (Figure 6; Table S3). Notably, the *ABAPT6* group has undergone the extensive loss of homologous genes across multiple dicot species (Figure 6). For example, the loss of *ABAPT6* is mainly found in species of Fabaceae (e.g., *Glycine max*) and Phrymaceae (e.g., *Mimulus guttatus*) species (Figure 6). A total of 96 duplications and 176 loss events within the *ABAPT5/6* branch were estimated in the reconciled gene tree using Notung 2.9. Furthermore, transcriptional profiles showed that some members of this group exhibited negligible or extremely low expression levels across nearly all assayed tissues, such as *ABAPT6* from *Eucalyptus grandis* and *Gossypium hirsutum*. This raises the question of whether these genes are pseudogenes without enzymatic activity or whether their mRNA accumulation depends on inducible expression under specific conditions (Figure 6; Table S7). Therefore, the above evidence indicated that the extensive gene gain and loss events have led to strong internal connections among members of the Cluster4-Monocot and further weakened their syntenic relationships with other members of the same subclade, thus ultimately driving the formation of this distinct cluster.

### 2.7. Expression Profiles of the ABAPT Genes and Identification of Pollen-Specific ABAPT Genes

**Figure 7:**
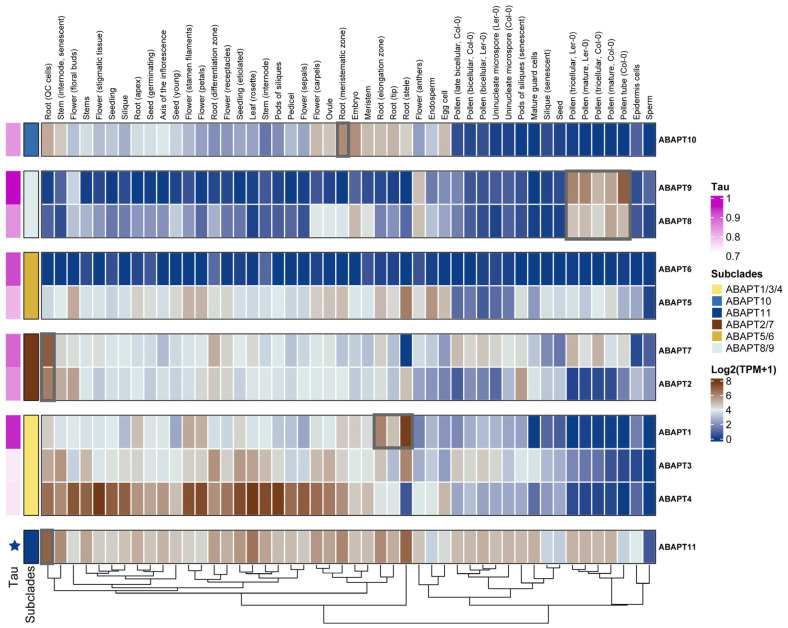
Expression analysis of 11 *AtABAPTs* in different tissues of Arabidopsis. The row cluster of genes corresponding to the *ABAPTs* in the phylogenetic tree is displayed together with the expression profiles, and the columns of tissues were split by k-means clustering in the ComplexHeatmap package (v1.0.2). The blue star represents a kind of broad expression pattern with a lower Tau value. Colors of cells indicate the abundance of transcription levels of ABAPTs in tissues. Transcript levels (TPM values) of all *AtABAPTs* are transformed into a log2(TPM+1) matrix. Cells with tissue-specific expression are highlighted in gray boxes. The heatmap is prepared using the CoNekT Export expression levels tool (https://evorepro.sbs.ntu.edu.sg/, accessed on 2 February 2025), and the source of expression data is available in Table S7.

To gain deep insights into the biological functions of the ABAPT gene family, we explored the expression patterns of *ABAPT* across seven species retrieved from the CoNekT database (https://conekt.sbs.ntu.edu.sg/, accessed on 2 February 2025), consisting of *O. sativa*, *Z. mays*, *A. thaliana*, *S. lycopersicum*, *A. trichopoda*, *Physcomitrium patens*, and *Ceratopteris richardii*. In addition, we subsequently used the TPM matrix to calculate the Tau index for each *ABAPT* gene (0 < Tau < 1). Our analysis revealed that the *ABHD13* clade exhibited relatively high levels and broad expression profiles, with a low Tau index across multiple organs like roots and leaves in all surveyed species (Figure 7 and Figures S11–S13; Table S7). In species with multiple *ABHD13* copies, such as soybean and cotton, one paralog exhibited a significantly higher expression than the other copy (Table S7). Given that recently duplicated gene pairs often show highly similar expression patterns, low-expression genes may lose function in the future due to rapid differentiation, and their overall expression may be restored to the single-copy level [35]. By contrast, a subset of *ABHD17* members exhibited relatively high Tau values, indicating a certain degree of tissue-specific expression (Figure 7 and Figures S11–S13). For example, in our investigated species, *ABAPT10* genes in Clade 2 were specifically expressed in the root, meristem, and ovule but showed low expression in reproductive organs like pollen and flowers (Figure 7 and Figures S11–S13). *ABAPT8/9*, the sister subclade of *ABAPT10*, exhibited pronounced expression in reproductive organs like pollen and pollen tubes. Furthermore, *ABAPT8s* were ubiquitously expressed in vegetative tissues like the stem in *Z.mays* and root in *A.thaliana*, whereas *ABAPT9* genes displayed tissue-specific expression almost exclusively restricted to pollen development; this suggested that the overall expression levels of the two duplicated genes are different after WGDs (Figure 7 and Figures S11–S13). In *Physcomitrium patens*, a model bryophyte species, the only two *ABHD17/13* homologs showed a relatively high expression in spore capsules, sporophytes, and paraphysis, highlighting their critical roles in sexual reproduction (Table S7). This tissue-specific expression may indicate functional conservation among genes within the same clade. For example, in Clade 3, *ABAPT1/3/4*s were ubiquitously expressed in most tissues, whereas most genes in the *ABAPT6* branch, such as *AtABAPT6* and *OsABAPT6*, were poorly expressed (TPM < 5). *ZmABAPT6* exhibited tissue-specific expression patterns in the root and stem but was undetectable in all other tissues (Figure 7 and Figures S11–S13). To gain additional insights into the pollen-specificity expression of *ABAPT8/9*, we extended our expression analysis of *ABAPT8/9* to *Amborella trichopoda*, an extant ancestor of angiosperms. *AmABAPT8/9* showed extremely high expression in male reproductive tissues, including pollen and pollen tubes (Table S7). Therefore, this expression reconstruction may reflect that the ancestor *ABAPT8/9* was likely highly expressed in male reproductive tissues, while during evolution, *ABAPT8/9* retained this ancestral expression pattern and maintained tissue specificity in plants. Additionally, we re-analyzed pollen development transcriptomics data in *O. sativa*, *Z. mays*, *A. thaliana*, and *S. lycopersicum*. *ABAPT8/9* genes were conclusively and specifically expressed during pollen grain development. For instance, *AtABAPT8* and *AtABAPT9* are cumulatively expressed during pollen maturation, reaching their highest expression levels in mature pollen and the pollen tube (Figure S14). Notably, *ABAPT8/9*s are abundantly expressed in pollen and the pollen tube in *Z. mays* and *O. sativa*, as well as in the developing pollen tubes of *S. lycopersicum* (Figure S14).

### 2.8. Gene Co-Expression Networks of ABAPTs and PATs Underlying Arabidopsis S-Acylation Cycle in Pollen Transcriptomics

**Figure 8:**
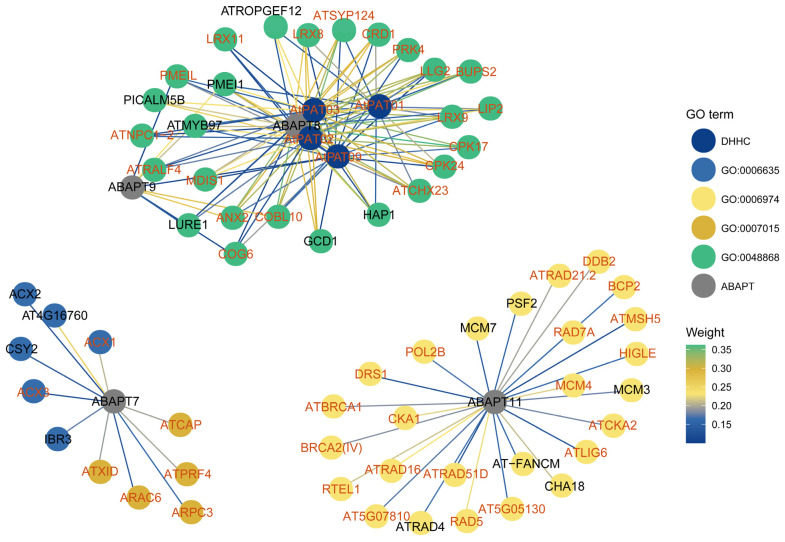
Networks of co-expressed genes in the modules containing *ABAPT7/8/9/11* and putative S-acylation partner *AtPAT1/2/3/9*. The colored dots of the legends on the right represent the corresponding GO terms that are significantly enriched with the *ABAPT* hub genes, respectively. GO:0006635, fatty acid beta-oxidation; GO:0007015, actin filament organization; GO:0006974, cellular response to DNA damage stimulus, and GO:0048868, pollen tube development. Genes in red have putative palmitoylation sites predicted by CSS-Palm 4.0 algorisms.

The functions of PATs and ABAPTs appear to be highly dependent on their auto-acylation to fulfill the S-acylation cycle involved in multiple signaling transduction pathways. Therefore, the complete and dynamically reversible catalytic cycle encompassing ABAPTs, target proteins, and PATs may underlie an intrinsic regulatory feedback mechanism [6,32,36]. Given that tissue-specific expression patterns are likely closely associated with the S-acylation process, we employed a traditional co-expression network approach to identify candidate regulatory modules [37,38]. Notably, the previously reported *AtPAT1*, *2*, *3*, *4*, *8*, *9* [39] and the *AtABAPT7/8/9/11* characterized in this study are predominantly tissue-specifically or highly expressed in pollen (Figure 7). As pollen development and pollen tube germination represent ideal model systems for investigating dynamic protein modifications that rapidly respond to external environmental changes and stimuli, we further analyzed the *ABAPT8/9* subgroup, which exhibits relatively conserved expression patterns. Weighted gene co-expression network analysis (WGCNA) was performed using the transcripts per million (TPM) matrix derived from the raw RNA-seq data of 75 pollen development expression profiles in *Arabidopsis thaliana* (pollen developmental stages for Ler and Col, accession: PRJEB39961), and we extracted co-expressed genes pertaining to *ABHD13*, *ABHD17*, and *PATs* from modules enriched for genes with similar expression patterns. Results showed that nearly all ABAPTs were assigned to the turquoise module, with the exception of *ABAPT11* (detected in the blue module). This observation suggested that *ABHD17* and *ABHD13* may exert distinct biological functions despite their overlapping expression profiles during pollen development (Figure 7 and Figure S14). Moreover, GO enrichment analysis revealed that the genes co-expressed with *ABAPT11* were significantly overrepresented in response to DNA damage stimulus, DNA repair, recombinational repair, and DNA replication (Figure 8; Table S8). This supports that de-S-acylation may also be required for DNA repair during meiotic progression in pollen mother cells, similar to the case of *AtPAT21* [40]. Gene ontology analysis demonstrated that genes associated with *ABAPT7* were significantly enriched in fatty acid metabolic processes. For example, *ABAPT7* was co-expressed with *ACX2*, which encodes an acyl-CoA oxidase presumably involved in long-chain fatty acid biosynthesis, and *ACX1*, catalyzing the first step of fatty acid beta-oxidation involved in jasmonate biosynthesis [41] (Figure 8; Table S9). Importantly, many co-expression partners of *ABAPT9* are typically related to pollen tube guidance and pollen development, such as *LIP2* and its paralog *LIP1*, which have been shown to interact with PENTAPAT (*AtPAT1*, *AtPAT2*, *AtPAT3*, *AtPAT4*, and *AtPAT8*) [37]. ANTH domain-containing proteins *PICALM5B* [42], *AtMYB97* [43], and *RALF4* [44], which are associated with pollen development and pollen tube guidance, represent promising candidate S-acylated substrate genes. Additionally, more than half of genes are co-expressed with *ABAPT8/9*, further implying their functional redundancy resulting from WGD events (Figure 8; Tables S10 and S11). Furthermore, most genes linked to *ABAPT* hubs were predicted to undergo S-acylation at a high-confidence threshold (Figure 8 and Tables S8–S11), providing underlying evidence for their potential role as S-acylated substrates.

## 3. Discussion

Over the past decade, research on protein palmitoylation and depalmitoylation in plants has addressed that this process not only serves as hydrophobic anchors for membrane association but also brings about profound changes to physicochemical properties and behaviors of target proteins [5,10,45]. However, research in this field is still in its infancy. With the increasing availability of sequenced plant genomics and proteomics, this area has attracted growing attention [5,45,46], thereby facilitating evolutionary studies and the characterization of plant ABAPT proteins.

### 3.1. Evolutionary Origins and Expansion of ABAPT Gene Family in Plants

A previous phylogenetic analysis of the *ABHD13* subfamily revealed an absence of homologs for this subfamily in bacteria. Notably, the earliest studies on this subfamily (*ABHD13*, bem46) were reported in eukaryotes as early as 2013 [47]. Findings of the *ABAPT* subfamily’s evolution from ancient archetypal genes have been exclusively derived from eukaryotes [15,16,47,48]. Regarding their origin, we infer that *ABAPT* first appeared after the emergence of eukaryotic life and is closely associated with their evolution. However, a more comprehensive analysis of early-diverging eukaryotic genomes needs to be conducted for clarification of the evolutionary origin of *ABAPT*, including representatives of basal protists (jakobids, diplomonads, and parabasalids), early branching fungi (chytrids, blastocladiomycetes, and mucoromycetes), and other phylogenetically deep eukaryotic lineages. This study provides clues about the origin of the *ABAPT* gene family in the common ancestor of algae—or even earlier organisms. Subsequently, massive lineage-specific gains and expansions of the *ABAPT* homologs have occurred in angiosperms (Figure 1; Table S1). The overall expansion of *ABAPTs* in plants is relatively modest and slightly lags behind that of *PATs* in other eukaryotic chlorophyte lineages [49]. This study also supports the independent evolution of *ABHD13* and *ABHD17* during land plant radiation, confirming that these two subfamilies were split apart from each other before the divergence of chlorophytes or even more ancient organisms (Figure 5, Figures S6 and S7). We cannot rule out the possibility that they do not share a common origin but were instead inherited from different ABHD families. This is supported by the general consensus regarding the ABHD superfamily, in which members that share a remarkably low sequence identity can also maintain a highly conserved role in the same catalyzed reactions [50,51]. In other words, traditional domain-based methods (e.g., HMM and BLAST searches) may not be suitable for identifying homologous relationships relevant to the origins of ABAPT. Further phylogenetic analysis of sectional and bulk ABAPT proteins revealed three main clades within the ABAPT family (Figure 3 and Figure 5). ABHD17s in the fern *Ceratopteris richardii* formed a sister phylogenetic branch with angiosperm Clade 3, implying that the remarkable expansion of Clade 3 could be traced back to fern diversification in the land plant lineage. Furthermore, all *ABHD17s* from primary green plants (including five mosses and one lycophyte) clustered together with Clade 2 in the phylogenetic tree, which represented a sister branch to angiosperm *ABAPT8/9* and *ABAPT10.* These findings not only support the ancient origin of Clade 2 in plants but also indicate that gene loss events have occurred in Clade 2 during fern evolution (Figure 3 and Figure 5).

Two main strategies were employed to infer phylogenetic relationships among *ABAPT* members. First, phylogenomic orthogroup inference methods (e.g., OrthoFinder2) were used to identify three orthologous groups (OGs) of ABAPT in 17 plants, which were consistent with the phylogenetic trees shown in Figure 3 and Figure 5. Second, synteny-based network analyses were able to complement existing homologous gene clustering methods (Figure 5). The syntenic signal in our network is in strong congruence with the observed phylogeny and supports the phylogenetic analyses of the ABAPT subfamily in angiosperms (Figure 5). This strategy was optimized to identify novel clades and distantly related homologous gene families for which phylogenetic methods alone may yield inconclusive or low-resolution results [21,25]. Phylogenetic and microsynteny network analyses revealed six well-supported *ABAPT* subclades, which complemented the results of the initial phylogenetic inference (Figure 3, Figure 5 and Figure S6). This synteny network further enabled us to identify multiple lineage-specific gene clusters. We characterized a major Cluster 4 community encompassing Poaceae-specific synteny, alongside several small syntenic communities, including a Fabidae-specific Cluster 10, as well as lineage-specific Cluster 9 in wheat and barley (Figure 5 and Figure S10). These genes, whose syntenic relationships were reconstituted through lineage-specific transposition and other gene duplication events, may differ from their homologs in terms of gene expression profiles and metabolic pathway regulation [22,33,52]. Notably, Cluster 4 originated from a shared WGD event in Poaceae plants (Figure 5 and Figure 6). Therefore, we speculate that these WGD-derived genes may be associated with agronomic traits of Poaceae, such as plant height and stem strength [28,53,54]. However, further research is necessary to dissect the role of the lineage-specific gene in Poaceae species’ agronomic traits.

A noteworthy finding is that a single copy of *ABHD13* was observed across diverse angiosperm lineages. Our results indicated that *ABHD17* contributed remarkably to the expansion of *ABAPT*-paralleled plant terrestrialization and land plant radiation (Figure 1; Table S1). Gene duplication analysis showed that three *ABHD17* copies have been preserved in *Sphagnum* lineages—representing the most ancient *ABAPT* expansion driven by two WGD events (189–247 Ma and 102–122 Ma; 95% CI) in the peat moss genus [30]. Considering the distribution of *ABHD17s* on chromosomes 6, 9, and 12 in *Sphagnum*, we speculate that the three *ABHD17* homologs are primarily derived from these two shared WGD events (Figure 2), with one copy lost on chromosome 7 [30]. Notably, phylogenomic synteny network analyses revealed that all six syntenic *ABAPT* subclades contain sequences from the pivotal basal angiosperm species, such as *Nymphaea colorata* and *Amborella trichopoda*, providing evidence of multiple ancient duplication events experienced before angiosperm occurrence (Figure S6). In conclusion, the *ABAPT* family first emerged in the algal genomes, subsequently diversified into two subtypes—*ABHD13* and *ABHD17*—and underwent dynamic gene expansion and loss events. Notably, this expansion was largely contributed by *ABHD17* duplications, particularly in members of Clade 3 (Figure 3 and Figure 5).

### 3.2. Characteristics and Possible Functions of ABHD13 in Plants

Duplicated genes can maintain ancestral functions or diverge through two evolutionary fates: subfunctionalization and neofunctionalization [35]. Phylogenetic delineation of *ABAPT* subfamilies resolved two distinct clades—ABHD13 and ABHD17—which exhibit substantial sequence divergence and potential functional diversification between these two subfamilies (Figure 5 and Figure 8). Moreover, further functional studies have provided additional evidence for protein substrate selectivity. Specifically, the selective removal of S-acyl groups from BSK1, BON1, and PBS1 by ABAPT11 is involved in the dynamic regulation of their subcellular localization. RPM1-Interacting Protein 4 (RIN4), a key component in pathogen resistance, is de-S-acylated exclusively by ABAPT8 [6,18,55]. However, the lack of information about large-scale de-S-acylation substrates has hampered studies on the role of de-S-acylation in the function divergence of ABHD13 and ABHD17, which may result in an important advancement in the field of plant S-acylation cycles. ABHD13s exhibit a distinctive structure comprising a conservative TMD region at the N-terminus, which is more hydrophobic than ABHD17 and may promote membrane binding through the hydrophobicity of the N-terminal helix (Figure 3). In Arabidopsis, ABHD13 proteins localize in the plasma membrane and membranes of inner membrane compartments rather than in the cytoplasm, nucleus, vacuoles, and apoplast [56]. Therefore, it is necessary to investigate further the N-terminal helix roles in ABHD13 activity to membrane subdomains, which may explain why ABHD13 appears to act primarily on substrates in the plasma membrane in the previous studies [6,18,55,57]. Unlike their animal homologs [58], plant ABHD13s contain the HX4D acyltransferase motif embedded within Motif 2—interspersed between the canonical GXSXG motif and core ABHD domain (Figure 3). Thus, these ABHD13 homologs may harbor dual activities as acyltransferase and de-S-acylation enzymes analogous to MAGLs [58,59].

Analysis of expression patterns indicated that *ABHD13s* are constitutively expressed at high levels in nearly all selected tissues (Figure 7 and Figures S11–S13; Table S7), which is similar to the animals’ *ABHD13* [58]. Therefore, *ABHD13* likely plays a widespread role in plant cell organization and function. This constitutive high-level expression also implies that *ABHD13* may be essential for the basic developmental and growth processes of cells across various plant tissues. Consistently, its pleiotropic roles have been proposed in root gravitropic responses, polarized morphogenesis in fungi, as well as BR-mediated growth and endocytosis-dependent modulated immune signaling in plants [15,18,55,56]. Previous reports also have revealed that both the transcript levels and protein abundance of ABHD13 are rapidly induced by salicylic acid treatment, highlighting its critical role in plant stress responses [18].

Unlike *ABHD17s*, *ABHD13*s have largely reverted to a single copy in angiosperms following recurrent rounds of paleo-polyploidy events like eudicot γ WGT and Poaceae ρ WGD (Figure S2). The retention of 2–4 duplicate genes of *ABHD13* is observed in species with recent polyploidy events. For example, duplicated *ABHD13s* have been retained as nearly identical gene copies that originate from the most recent WGD event (13 million years ago) shared by all *Glycine* genera [27]. Therefore, ABHD13 may represent a housekeeping gene that is not a substrate-specific enzyme and can de-S-acylate a wide range of substrates irrespective of tissue types, thereby fulfilling extensive and conserved biological roles in plants. Such modification may represent a fundamental step for the proper functioning of many proteins. For example, ABAPT11 is involved in the de-S-acylation of two proteins: BSK1, which regulates the BR signaling pathway, and BON1, which orchestrates the development–immunity balance via endocytosis [32,55]. Examples in these studies may be just the tip of the iceberg.

### 3.3. Characteristics and Possible Functions of ABHD17 in Plants

Most ABHD17 and ABHD13 proteins only share approximately 20–30% sequence identity, implying potential functional divergence between these two protein subfamilies. Moreover, the ancient *ABHD17* genes underwent multiple duplication events before the radiation of angiosperms and further diverged into two distinct clades, Clade 2 and Clade 3 (Figure 5 and Figure S6). Clade 2 then further diversified into two separate subclades, including *ABAPT8/9* and *ABAPT10*. Phylogenetic reconstruction showed that all moss-derived *ABHD17s* clustered together with angiosperms *ABAPT8/9* and *ABAPT10*, which maintained remarkably conserved exon/intron structures with mosses (Figure 3 and Figure 5). Taken together, the conservation of these feathers in this respective clade may represent the earliest *ABHD17* members after the separation of mosses. ABAPT10 in Clade 2 differs from other ABHD17 homologs in its unique C-terminal region containing a cysteine-rich (Cys-rich) region. Notably, it is distinctly different from its sister group ABAPT8/9 in terms of length and domain structure (Figure 3). Interestingly, we concluded that the cysteine-rich region was required for the membrane localization of ABAPT10 and hypothesized that the cysteine-rich region of ABAPT10 may exert effects on their PM delivery [60]. However, we did not observe vesicle-like clustering structures in the mutation of ABAPT10^C3/196/197/251/348/352/353/354S^. Therefore, we also speculate that some putative S-acylation sites of the cysteine-rich region were underestimated or regulated by other post-translational modifications.

The largest clade, Clade 3, was further separated into three distinct groups. The weak synteny interconnections among these three subclades imply a shared genomic history and potential functional relationships among their members (Figure 5 and Figure S6). In contrast to Clade 2, the presence of one to four C-terminal Motif 8 repeats was identified as a key feature specifically enriched in Clade 3 (Figure 3). Moreover, Motif 14 corresponds to the C-terminal segment and was unique to the ABAPT5/6 subclade (Figure 3). The disordered regions of the C-terminal containing conserved motifs were defined as IDRs in Clade 3, providing numerous valuable targets for future investigations. For example, the binding promiscuity and plasticity conferred by IDRs may facilitate target recognition by their de-S-acylation protein, which may allow the limited number of de-S-acylation enzymes in plants to perform the de-S-acylation on a broad spectrum of substrates [61,62]. Moreover, a link between disorder and phosphorylation has been elucidated for the Remorin family, which binds to the plasma membrane via the conserved C-terminal region [31,32,63]. This raises the question of whether the phosphorylation of ABAPTs by unknown substrate kinases is facilitated by their IDRs. On the other hand, S-acylation frequently occurs in predicted disordered regions or within/adjacent to α-helices, which may be critical for their auto-acylation activity and substrate specificity [64].

### 3.4. ABAPT8/9 May Be Co-Evolved with the Pollen-Specific PAT1/2/3/9

The dynamic S-acylation cycle (PAT–substrate–ABAPT/APT) allows plants to rapidly adapt to external environmental changes and thus improves their stress resilience [36,55,65]. Previous phylogenetic analyses have depicted that *AtPAT1*, *AtPAT2*, *AtPAT3*, *AtPAT4*, *AtPAT8*, and *AtPAT9* belong to Group A with a rather high conservation rate. Among these paralogs, *AtPAT2*, *AtPAT3*, and *AtPAT8* were specifically enriched in stamens and pollen [39,49]. A few PAT–substrate and APT–substrate pairs have been experimentally demonstrated and functionally linked to pollen tube guidance in plants. For example, PAT-mediated S-acylation is vital for rapid pollen tube germination and micropylar guidance in *Arabidopsis thaliana* [66]. S-acylation of PRK1 is essential for pollen tube guidance through modulating PM receptor complexes [37]. These findings indicate that essential molecules involved in pollen function may undergo lipid modification by both PATs and ABAPTs, and the S-acylation cycle is presumably implicated in pollen development as well. However, the example of proteins that actually undergo S-acylation cycling during pollen development remains largely unknown to date. Likewise, the functional cooperation and co-evolution between PATs and ABAPTs still require validation from further experimental and bioinformatic evidence, such as evolutionary rate covariation (ERC) analysis and pair-wise interaction between PATs and ABAPTs [67,68]. In this study, we characterized the expression profiles of *ABAPT* family genes and found that *AtABAPT8/9*s are also predominantly or specifically expressed in pollen grains (Figure 7 and Figure S14). Co-expression networks provide valuable insights into the complex gene–product interactions and are useful for PPI prediction [69]. Using a co-expression network, we identified several potential functional modules, including *AtPAT1/2/3*-*LIP1/2*-*ABAPT8*, *AtPAT1/2/3/9*-*ATSYP124*-*ABAPT8*, *AtPAT2/3/9*-*MDIS1*-*ABAPT8*, *AtPAT2/3/9*-*ANX2*-*ABAPT8/9*, and *AtPAT1/2/3/9*-*LLG2*-*ABAPT8*. We also predicted several putative S-acylation sites in these proteins using CSS-Palm 4.0 (Figure 8; Table S12). Notably, LIP1/2, the only functionally characterized substrates in our network, provide valuable clues for understanding the regulatory roles of the S-acylation cycle in plant reproduction [66]. The functional mechanisms underlying these putative S-acylation cycle modules during pollen polar growth and fertilization remain to be further elucidated.

## 4. Materials and Methods

### 4.1. Plant Materials and Growth Conditions

Seeds of *Nicotiana benthamiana* and *Arabidopsis thaliana* (Col-0) were incubated at 4 °C for three days and surface-sterilized in 75% (*v*/*v*) ethanol for 30 s and 10% (*v*/*v*) bleach for five min, then the seeds were rinsed thoroughly with sterile water and plated on half-strength MS medium with 1% (*w*/*v*) agar. *N. benthamiana* was cultivated in a growth room at 25 °C with 65% relative humidity under a 12 h light/12 h dark photoperiod (normal growth conditions). Seeds of Arabidopsis were then moved to 21 °C under continuous light for germination for one week, then seedlings were transferred to the soil under long-day conditions (16 h day/8 h night) at 21 °C. Four–five-week-old *N. benthamiana* seedlings were collected prior to sample treatment.

### 4.2. Subcellular Localization of AtABAPT2 and AtABAPT10

Total RNA was prepared from Arabidopsis 7-day-old seedlings using the RNeasy Plant Mini kit (Qiagen, Valencia, CA, USA). The first-strand cDNA was produced from 2 mg of RNA using the RevertAid First Strand cDNA Synthesis kit (K1621, Thermo Scientific, Waltham, MA, USA) with oligo(dT)12-18 primer according to the kit’s instructions. The full-length coding sequences of *AtABAPT2* and *AtABAPT10* were amplified using forward and reverse primers (listed in Table S13) with *BamH*I and *Sal*I restriction sites and cloned into pCAMBIAsuper1300 vector using the In-Fusion^®^ Snap Assembly Kit (Takara, Shiga, Japan). Site-directed mutagenesis was conducted using Mut Express II Fast Mutagenesis Kit V2 (Vazyme, C214-01, Nanjing, China). Briefly, fragments overlapping at the mutation sites were amplified and the PCR products were digested with *Dpn*I for 2 h at 37 °C, and 5 μL thereof was used to transform the *E. coli* strain DH5α. The recombinant vectors were then transformed into *Agrobacterium tumefaciens* strain GV3101 via the freeze–thaw method. *A.tumefaciens* strains harboring the resultant 35S::*ABAPT2*-eGFP, 35S::*ABAPT10*-eGFP, 35S::*ABAPT2*-2C2S-eGFP, 35S::*ABAPT10*-8C2S-eGFP, and plasma membrane (PM) marker 35S::*AtPIP2*-mCherry were mixed and co-infiltrated into the abaxial side of tobacco leaves using 1 mL syringe without needle. The infiltration buffer contained 10 mM MES/NaOH, 10 mM MgCl_2_, and 600 μM acetosyringone (pH 5.6), and the final OD_600_ of strain solution was adjusted to 1.0 and incubated at room temperature without agitation for 2–3 h. Infiltrated seedlings were incubated under the same growth conditions for an additional 2–3 days. Fluorescence signals were observed using confocal scanning microscope (Leica SP8, Wetzlar, Germany), with excitation wavelengths of 488 nm for eGFP and 587 nm for mCherry. The number of circulating vesicles was quantified using an automated analysis module in Fiji (v2). This experiment was carried out with three biological replicates, each sample consisting of at least 10 cells was analyzed, and the statistical analyses included a cut-off value of *p* < 0.01, paired *t* test, and asymptotic *p*-value computation (implemented in the R package, ggpubr, version 0.6.3).

### 4.3. Identification of ABAPT HMMERlogs in Sequenced Plant Genomes

To perform a broad-scale *ABAPT* family survey and ensure a wide spectrum of sampled taxonomic phylogeny, we collected peptides, CDS sequences, and GFF3 annotation files from multiple comprehensive databases, including Phytozome (https://phytozome-next.jgi.doe.gov/, accessed on 2 May 2023) and Ensembl (https://www.ensembl.org/, accessed on 2 May 2023). The analyzed species of all sources are listed in Table S1. To identify putative sequences encoded by ABAPT from each genome database using the available annotated peptide files, the 11 *Arabidopsis* ABAPT proteins were used as queries against corresponding plant proteomes using local BLASTp (v2.4.10) with the following parameters: -max_target_seqs 10, -evalue 1 × 10^−20^. Additionally, HMM profiles of ABAPT-related domain were downloaded from ESTER database (https://bioweb.supagro.inra.fr/esther, accessed on 2 May 2023). To fine-tune the subfamily discrimination process, HMM profiles specific to ABHD13/17 were isolated for the searches using -E < 1 × 10^−15^, --domE < 1 × 10^−15^ as default parameters. Subsequently, HMM profiles for ABHD13 and ABHD17 were rebuilt using aforementioned candidate genes combined with BLASTp and hmmsearch and used to analyze the conserved domains of putative ABAPT proteins across plant lineages. The cut-off E-value and domain E-value = 1 × 10^−30^ and the corresponding HMM library were chosen for the delineation of the final subfamilies in ABHD17, and sequences with more than 95% can be considered to definitively belong to ABHD17 subfamily. Redundant sequences (e.g., those arising from alternative splicing, short sequence lengths, are listed in Table S1) were removed using the GXF module of TBtools (v2.0) and the remaining sequences were manually inspected for genome annotation errors or multiple stop codons [70]. Finally, the conserved domain of all deduced ABAPT protein sequences was verified via the Interproscan and Pfam database. To avoid confusion, the names of 11 Arabidopsis ABAPT proteins in the previous study were retained here [6].

### 4.4. Multiple Sequence Alignment and Phylogenetic Analysis

Multiple sequence alignment of the identified ABAPTs was conducted by MUSCLE (implemented in MEGA11 program, version 5) with default parameters. TrimAl (v2.rev0) was then employed to remove spurious sequences or poorly aligned regions with the automated1 option, which applies a heuristic to decide the optimal method for alignment trimming. Then, the trimmed sequences were subsequently submitted to IQ-TREE (v2.4.0) for phylogenetic tree construction with the following parameters: -m MFP -T 10 -B 10000 --bnni --alrt 10000. The ModelFinder was used to determine the best-fit model and Bayesian information criterion (BIC) was used to evaluate the log-likelihoods of an initial parsimony tree for many different models (the model with the minimum BIC value as the substitution models). The standard nonparametric bootstrap was invoked with 1000 bootstrap replicates for phylogeny reconstruction of *ABAPT5/6*. MrBayes (v3.2) was performed for the Bayesian inference of phylogeny using 10,000,000 generations, ensuring that two runs were sufficient to reach convergence, and the first 25% of the sampled trees from two runs was discarded as burn-in with default. Bayesian MCMC analysis is acceptable when the standard deviation of split frequencies is below 0.01. Phylogenetic trees were visualized using ggtree (v4.0.4) and ggtreeExtra (v1.20.1) in the R statistical environment (v4.4.2). Phylogenetic relations and divergence times of species were estimated using Timetree database (https://timetree.org/, accessed on 2 September 2025).

### 4.5. Extraction of Synteny Network and Detection of Syntelog Community

Phylogenomic profiling, gene synteny, and collinearity analyses were performed using Syntenet (v1.8.0) [71]. We identified intra- and interspecies syntenic blocks of the 59 plant species using standard programs (BLAST (v2.4.10) and MCScanX (v1.0.0), anchors = 5, max_gaps = 25). Infomap algorithm—a fast community detection method implemented in the Syntenet (v1.12.0)—was used to detect syntelog communities [23,71]. The ‘plot_profiles’ function was used to visualize phylogenomic profiles as a heatmap, with species displayed in rows and synteny network clusters in columns. Synteny network of the *ABAPT* gene family was generated and visualized using Fruchterman Reingold layout algorithm implemented in Gephi (v0.9.2). Communities (synteny clusters) derived from Infomap algorithm were extracted, the node (i.e., gene) and edge (i.e., syntelog) compositions of each community were then mapped to the phylogenetic tree using ggtree (v4.0.4) and ggtreeExtra (v1.20.1).

### 4.6. Analysis and Visualization of Microsynteny

Local gene content was explored by JCVI (v1.4.22) package (Python3, module: ‘jcvi.compara.synteny’), in which flanking genes encompassed the *ABHD13*-centric blocks. The command line option ‘--iter=1’ was adopted to extract one best region matching each *ABHD13* region. Subsequently, local synteny plots were generated using the ‘jcvi.graphics.synteny’ function.

### 4.7. Analyses of Gene Structure and Gene Duplication Types

Intron/exon structures and intron phases were extracted and determined using gffread (v0.12.7) and visualized with CFVisual (v2.1). Potential transmembrane domains within ABAPT proteins were predicted using the online server DeepTMHMM (https://dtu.biolib.com/DeepTMHMM/, accessed on 30 May 2024). CSS-Palm (v4.0) was adopted to perform the potential lipidation site prediction of ABAPTs with default thresholds. The MobiDB-lite tools (https://mobidb.org/, accessed on 30 May 2024) and DisProt (https://www.disprot.org/, accessed on 30 May 2024) were used for examining intrinsic disorder regions (IDRs). To identify conserved motifs, we combined representative sequences from ABHD13 and ABHD17 subfamilies’ proteins and scanned them using MEME (v5.5.9) with default parameters, except for motif site distribution; the -anr (anr = “any number of repeats”) mode was selected instead of -oop or -zoops to allow for detection of motifs that repeat multiple times within a single sequence. Sequence logos plot was supported by ggseqlogo package (v0.2.2). Standard procedures for gene duplication mode classification of DupGen_finder (https://github.com/qiao-xin/DupGen_finder, accessed on 10 May 2023) were followed as a guide pipeline, of which five modes of gene duplication can be defined in this Perl scripts scheme [26,29]. For detecting the transposition-derived duplication, basal angiosperm *Amborella trichopoda* and *Nelumbo nucifera* were selected as outgroups for ancestral syntenic block anchors. The ‘DupGen_finder-unique.pl’ script was used to eliminate redundant duplicate genes among different modes in which a gene will be assigned a unique duplication model corresponding with the followed priority: WGD > tandem > proximal > transposed > dispersed.

### 4.8. Calculations and Distributions of Nonsynonymous (Ka) and Synonymous (Ks) Nucleotide Substitution Rates

Putative orthogroups from species representing major lineages of green plants were retrieved using OrthoFinder v2.5.5 algorithm with the following parameters: -S diamond -M msa -A mafft -os -T fasttree. We used the ‘pairs2kaks’ function implemented in the R package doubletrouble (v1.10.0) to calculate Ka and Ks values with at least two codon substitution models (YN and NG methods) [29]. To ensure reliable timing of duplication events, only gene pairs originating from whole-genome duplication were retained as evidence for WGD events. Due to over-clustering of peaks (i.e., prediction of more peaks than the actual number) in mixture modeling techniques (e.g., Gaussian mixture models), we referred to previously published studies to determine the numbers of polyploidization events and then identified peaks in the Ks distribution using the ‘find_ks_peaks’ function. The significance of each peak was tested using SiZer (v0.1-8, Significant ZERo crossings of derivatives) maps implemented in the R package ‘feature (v1.2.15)’. Histograms of Ks distributions with peaks and *ABAPT*-related blocks were generated by ‘plot_ks_peaks’ function and ggplot2 (v4.0.0).

### 4.9. Transcriptome Analysis and WGCNA Co-Expression Network Construction

Gene expression levels of *Arabidopsis thaliana* (Col-0) pollen development stages, including uninucleate microspores, bicellular pollen, late bicellular pollen, mature pollen, and pollinated pistil, were quantified using public source of RNA-seq data deposited in NCBI Sequence Read Archive (SRA). Each sample contained tissue from 6 individuals in two biological replicates. All samples described were filtered, aligned, and quantified using the same software, and parameters as indicated below. Briefly, raw reads were assessed and filtered by FastQC (v0.12) and Trimmomatic (v0.40) [72]. The ‘infer_experiment.py’ script module from RSeQC package (v5.0.0) was used to determine the RNA-seq sequencing configuration for downstream read count analysis. After quality trimming and adapter removal, the remaining clean reads were mapped to their respective reference genomes with HISAT2 (v2.2.1) and quantified for gene expression by HTSeq (v2.0.5) with user-defined defaults. TPM and FPKM values for each gene were calculated with Perl scripts ‘counts2tpm.gtf’ and ‘counts2fpkm.gtf’ (https://github.com/wfaf019/count2TPM, accessed on 1 May 2024). WGCNA was performed for the individual gene expression dataset of each genotype using the functions in R package WGCNA (v1.72-5).

## Data Availability

The original contributions presented in the study are included in the article/Supplementary Materials. Further inquiries can be directed to the corresponding authors.

## Supplementary Materials

The following supporting information can be downloaded at: https://www.mdpi.com/article/10.3390/ijms27083691/s1.
