# Supplementary material for: Phylogenomic Insights into High Conservation and Lineage-Specific Expansion of the ABAPT Gene Family in Plants
Source: Int J Mol Sci. 2026 Apr 21;27(8):3691. doi: 10.3390/ijms27083691 (PMC13116537; doi:10.3390/ijms27083691)
Supplement: Supplementary file 1 [file ijms-27-03691-s001.zip › Legend of Supplement Figures.pdf]

Supporting Information

Supplementary Figures

Figure S1. Sequence conservation of ABAPT and DHHC-domain PATs in plants.
Sequence conservation of DHHCs and ABAPTs in **(A)** *Arabidopsis thaliana* (At); **(B)** *Brassica oleracea* (Bol); **(C)** *Brachypodium distachyon* (BradI); **(D)** *Brassica rapa* (Bara). Sequence identity percentages of all protein pairs are determined, and the distribution (as frequency) of sequence identity scores is plotted as a histogram. The vertical line denotes the mean sequence identity value of each gene family.

Figure S2. Microsynteny relationships between the *ABHD13*-containing scaffolds, or their associated genomic blocks in representative plant genomes.
Neighboring genomic regions of the *ABHD13* locus in all genomes are syntenic to another genomic block where *ABHD13* genes were probably lost. Different box in the same color represents the homologous gene pairs. *ABHD13* loci are highlighted by a red box with gene ID shown above. Syntenic flanking genes connected by gray curves. The plant species are depicted by their abbreviated scientific names: Athaliana, *Arabidopsis thaliana*; Fvesca, *Fragaria vesca*; Bvulgarisssp, *Beta vulgaris*; Phallii, *Panicum hallii*; Carietinum, *Cicer arietinum*; Qrubra, *Quercus rubra*; Dalata, *Dioscorea alata*; Sbicolor, *Sorghum bicolor*.

Figure S3. Collinearity of the *ABHD13*-associated genomic blocks in basal eudicot *V.vinifera*.
Genomic synteny of adjacent genes of *ABHD13* loci (*VIT_211s0016g01490*, red box) was investigated in grapevine genome (*Vitis vinifera*), which has not undergone recent genome duplication after the whole-genome triplication restricted to core eudicots. Different box in the same color represents the homologous gene pairs. Syntenic flanking genes connected by gray curves.

Figure S4. Phylogenetic analysis of ABAPTs in representative species from various plant lineages.
Maximum likelihood phylogenetic tree of the ABAPT family across 17 species was constructed using IQ-TREE2 under the Q.plant+R4 substitution model. Bootstrap support values (from 1000 replicates) are indicated at each node. Arabidopsis TIPSY1 (AT4G22300, carboxylesterases) was selected as an outgroup, as in a previous report [6]. ABHD17s from moss that are closely related to ABAPT8/9 and ABAPT10 are marked with a yellow-brown branch. Homologous ABAPT proteins from Arabidopsis are highlighted in red.

Figure S5. The C-terminus residues are involved in the plasma membrane association of ABAPT10.
(A) Vesicle-like structures (white arrowheads) that were not associated with the plasma membrane remained unaltered throughout the imaging period. Images were acquired at 2-minute intervals. **(B, C)** The overlapping fluorescence spectra of the 35S::AtPIP2-mCherry and 35S::ABAPT10-1-272-eGFP signal intensities were analyzed along each white dashed line and plotted at 0 min **(B)** and 10 min **(C)**. Scale bar: 25 μm.

Figure S6. Synteny network of the *ABAPT* family members, constructed using synteny network database of angiosperms.
Community assignments were generated based on the Infomap algorithm as implemented in the R package *igraph*, which assigns each gene to a single cluster. Nodes highlighted in red indicate basal angiosperm (*Amborella trichopoda*), which harbors multiple ancient archetypal *ABAPT* types. Node colors correspond to *ABAPT* subclades, consistent with the classification shown in Figure 5B.

Figure S7. Synteny network of four clusters from three charophytes, four bryophytes, one lycophyte and one fern.
All sequences are indicated by five- or two-letter acronyms preceding gene names. Synteny network of *ABAPT* was constructed from nine non-seed plants including three algae, four mosses, one lycophyte (*Diphasiastrum complanatum*) and one fern (*Ceratopteris richardii*). Abbreviations for plants: Cepur; *Ceratodon purpureus*, Dicom; *Diphasiastrum complanatum*, Pp; *Physcomitrium patens*, Sphfa; *Sphagnum fallax*, Sphma; *Sphagnum magellanicum*.

Figure S8. Syntenic and evolution relationships between the *ABHD17s* from Arabidopsis and *Solanum tuberosum* within Clade 2 and Clade 3.
(A) Gene trees of Clade 2 and Clade 3 were constructed by maximum-likelihood method with 1,000 bootstrap replicates, and different symbols represented various species. Genes highlighted with light blue shading correspond to the syntenic relationships presented in Figure 5B. The phylogenetic trees are rooted by ABAPT from *Ceratodon purpureus* for clade delineation.
(B) Local syntenic relationships in Clade 2 and Clade 3 are further confirmed with jcvi microsynteny visualization. The particular syntelogs in the Figure 5B are highlighted with red curves. Abbreviations for plants: Ath, *Arabidopsis thaliana*; Stu, *Solanum tuberosum*.

Figure S9. Syntenic and evolution relationships between *ABHD17s* from *Cicer arietinum* and other Fabidae lineage within Clade 2 and Clade 3.
(A) Gene trees of Clade 2 and Clade 3 were constructed by maximum-likelihood with 1,000 Bootstrap replicates, and different symbols represent various species. Genes highlighted with light blue shading correspond to the syntenic relationships presented in Figure 5B. The phylogenetic trees are rooted by ABAPT from *Ceratodon purpureus* for clade delineation.
(B) Local syntenic relationships in Clade 2 and Clade 3 are further confirmed with jcvi microsynteny visualization. The particular syntelogs in the Figure 5B are highlighted with red curves. Our analysis suggests distinct subclades for syntelogs in some species. The observed phylogeny exhibits a marked incongruence with this syntenic signal. Abbreviations for plants: Car, *Cicer arietinum*; Cil, *Carya illinoinensis*; Gma, *Glycine max*; Ler, *Lens ervoides*; Pac, *Phaseolus acutifolius*; Qru, *Quercus rubra*.

Figure S10. Phylogenetic profiling of 10 network clusters obtained by Infomap from *ABAPTs* across 50 plant species.

Colors of cells depict the numbers of syntelogs in different species. Species names are shown on the right side, and the color bar on the left of heatmap represents the family ascription of species. Lineage-specific communities are highlighted by the dashed framework. Numbers at the bottom of cells denote the community IDs detected by R igraph using the Infomap algorithm.

Figure S11. Expression analysis of *ABAPTs* in *Oryza sativa*.

Colors of cells indicate the abundance of *ABAPT* transcription levels in tissues, while grey box of cells indicates abundant transcription in preferred tissues. The blue star represents a broad expression pattern with lower Tau value in *OsABHD13*.

Figure S12. Expression analysis of *ABAPTs* in *Zea mays*.

Colors of cells indicate the abundance of *ABAPT* transcription levels in tissues, while grey box of cells indicates abundant transcription in preferred tissues. The blue star represents a broad expression pattern with lower Tau value in *ZmABHD13*.

Figure S13. Expression analysis of *ABAPTs* genes in *Solanum lycopersicum*.

Colors of cells indicate the abundance of *ABAPT* transcription levels in tissues, while grey box of cells indicates abundant transcription in preferred tissues. The blue star represents a broad expression pattern with lower Tau value in *SolABHD13*.

Figure S14. Heatmaps of transcription level at different stages of male gametophyte development.

(A) Heatmap plot of expression levels of 6 *ABAPTs* and *21 PATs* obtained by RNA-seq analysis of the Arabidopsis pollen transcriptome (Pollen developmental stages for Ler and Col, Accession: PRJEB39961). Development stages of Arabidopsis male gametophyte can be divided into pollen mitosis I and II, which produce bicellular and tricellular pollen, respectively. After pollination of a receptive stigma, pollen grains germinate to grow pollen tube for double fertilisation. The hub genes, which are involved co-expression partner between *AtPATs* and *AtABAPTs*, are marked in red text.

(B) Expression profiles of *OsABAPTs* genes in different tissues (Mature pollen, anther at stage7-8, and seed endosperm of 25 days after pollination) retrieved from NCBI (Accession: PRJNA557342, accessed on 20 March 2025). R1 and R2 are biological replicates.

(C) Heatmap of expression profiles of *SlABAPTs* during pollen tube dynamic development of *Solanum lycopersicum*. R1, R2 and R3 are biological replicates. Transcription data were obtained from NCBI (Accession: PRJEB41071, accessed on 22 March 2025) and reanalyzed.

(D) Heatmap of expression profiles of *ZmABAPTs* in core male tissues of *Zea mays*. The tissues are mature pollen and pollen tube. R1, R2 and R3 are biological replicates. Transcription data were obtained from NCBI (Accession: PRJEB40911, accessed on 24 March 2025) and reanalyzed. The genes with TPM (Transcripts PerKilobase of exon model per Million mapped reads) value ≥ 5 in at least one tissue were used to ensure the reliability of the gene expression patterns.
