# Supplementary figures and images for: Phylogenomic Insights into High Conservation and Lineage-Specific Expansion of the ABAPT Gene Family in Plants
Source: Int J Mol Sci. 2026 Apr 21;27(8):3691. doi: 10.3390/ijms27083691 (PMC13116537; doi:10.3390/ijms27083691)

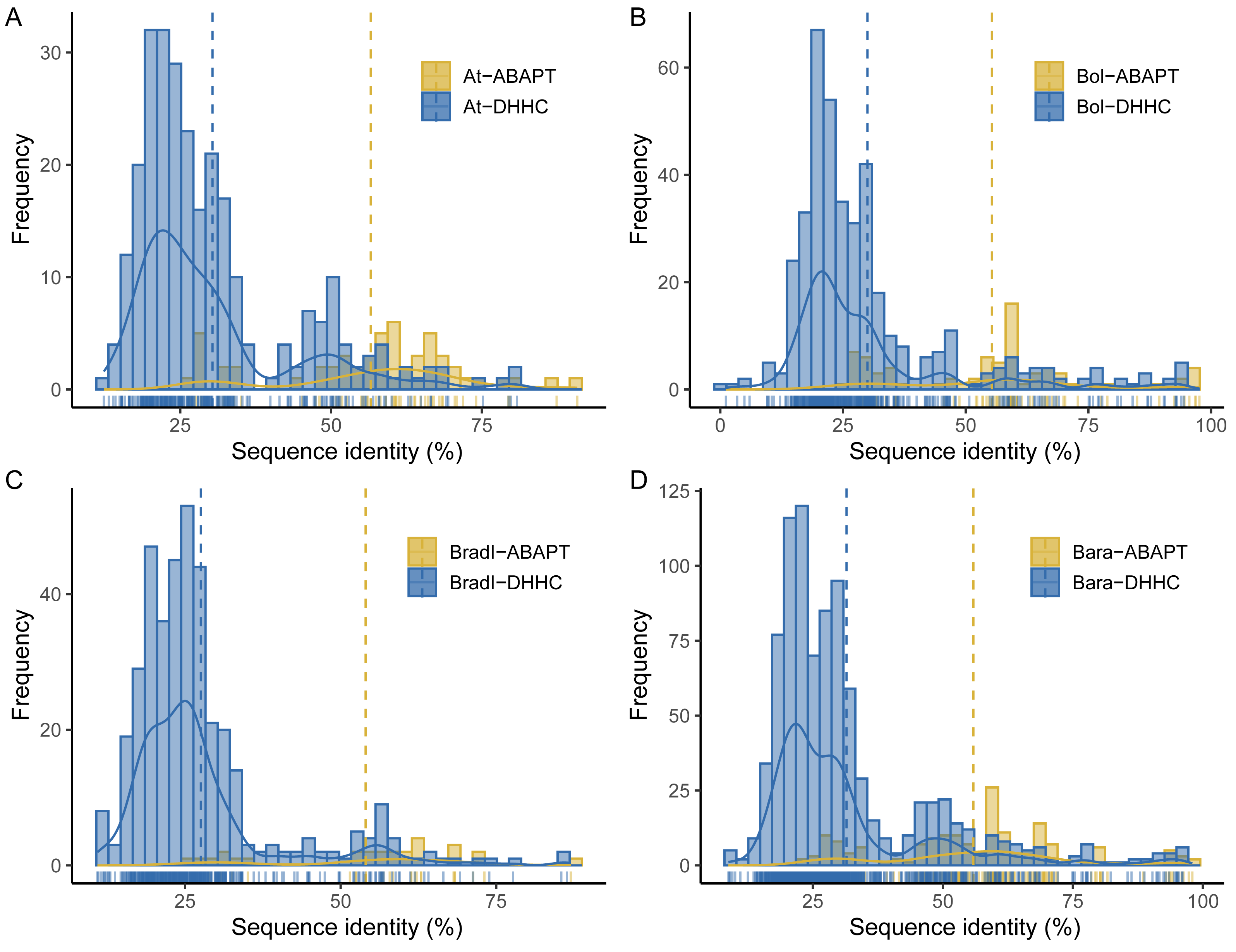

Supplement: Supplementary file 1 [file ijms-27-03691-s001.zip › Supplement Figures/Figure S1.tif]

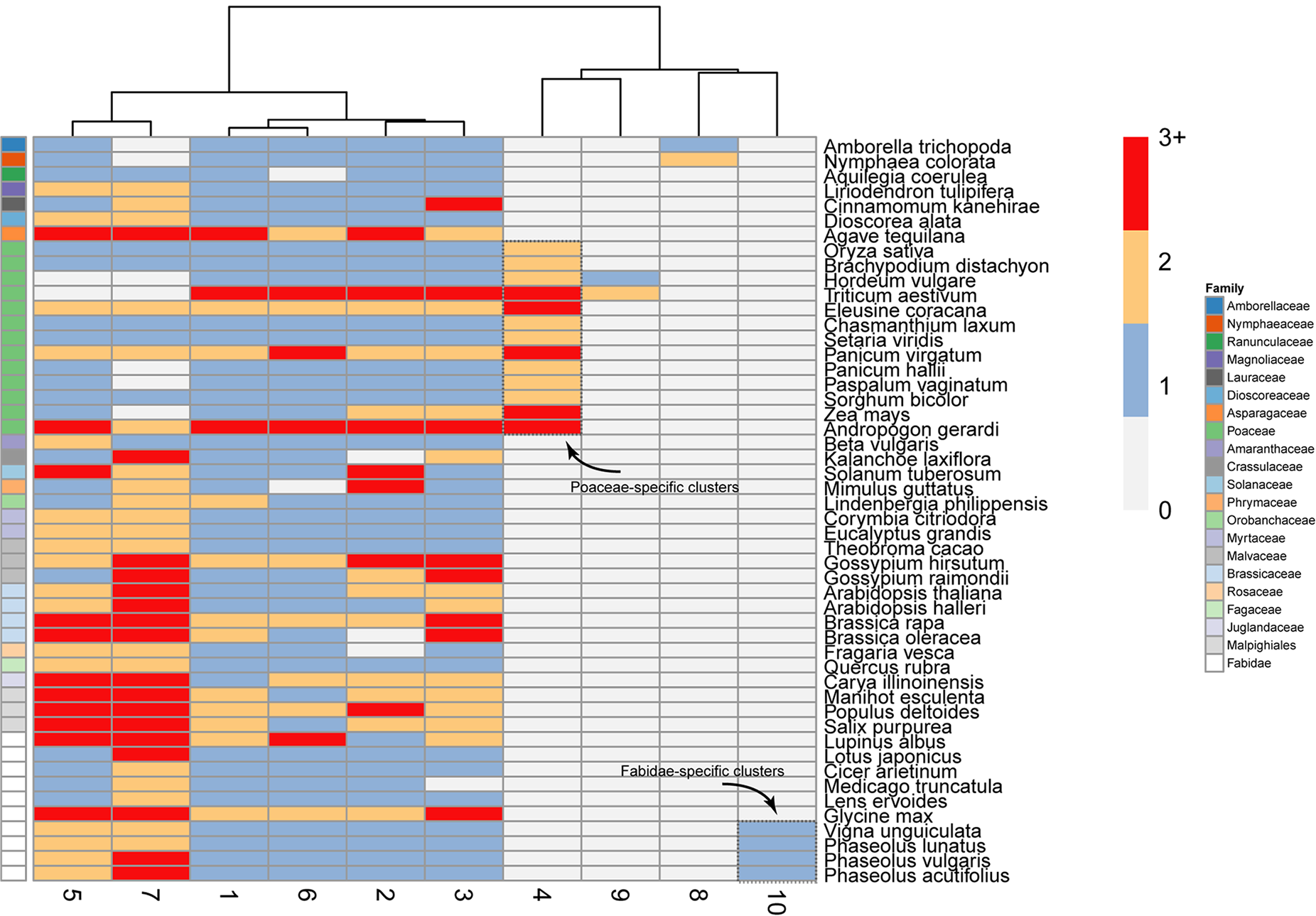

Supplement: Supplementary file 1 [file ijms-27-03691-s001.zip › Supplement Figures/Figure S10.tif]

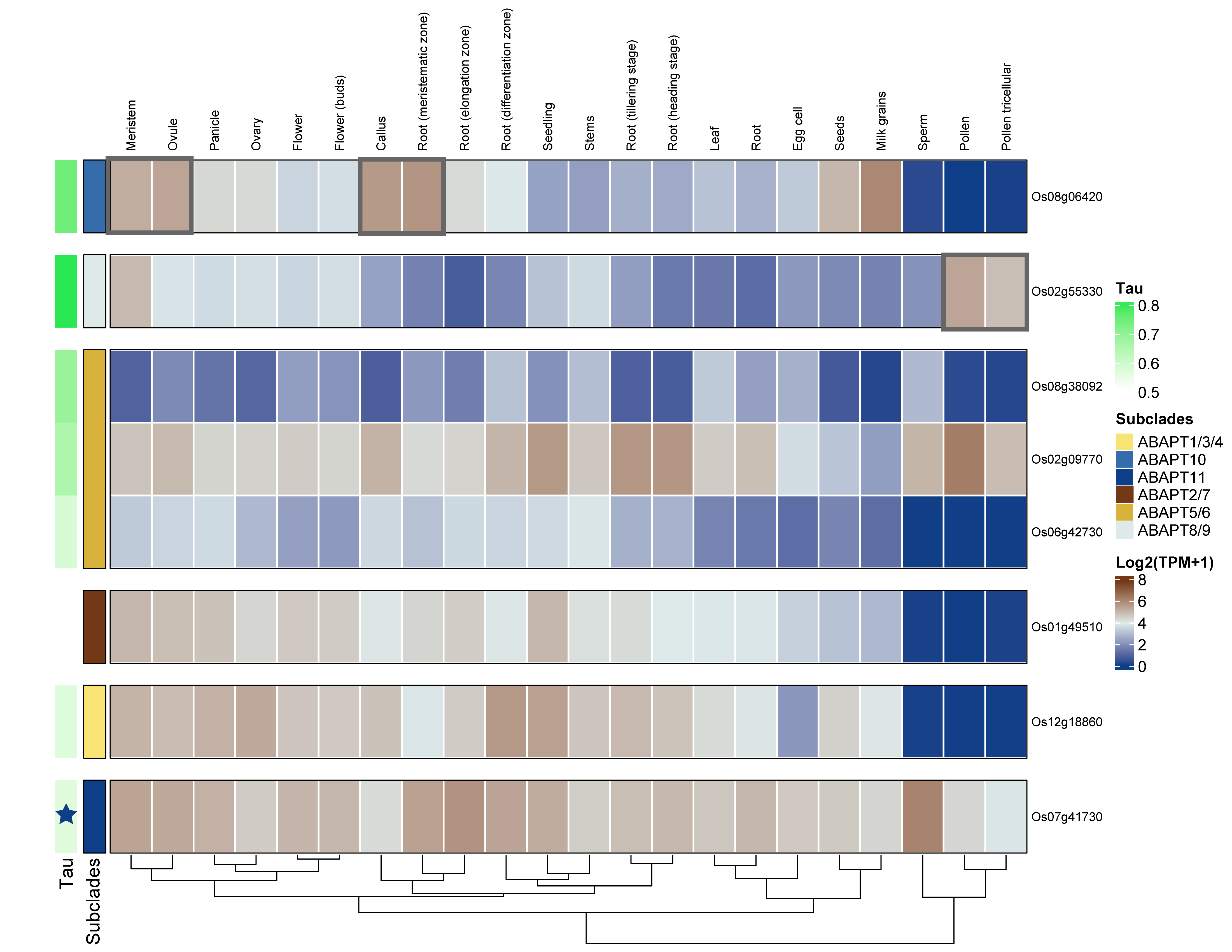

Supplement: Supplementary file 1 [file ijms-27-03691-s001.zip › Supplement Figures/Figure S11.tif]

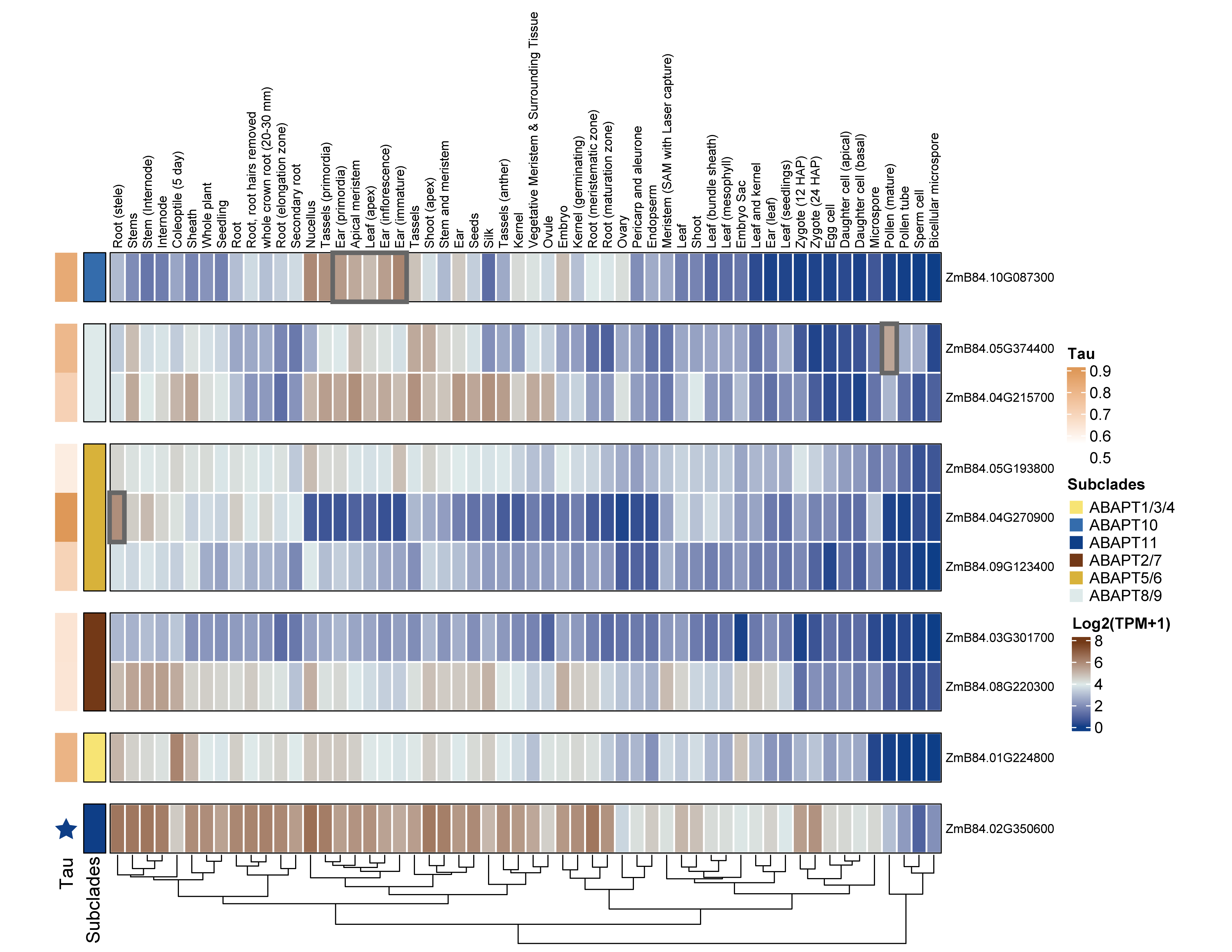

Supplement: Supplementary file 1 [file ijms-27-03691-s001.zip › Supplement Figures/Figure S12.tif]

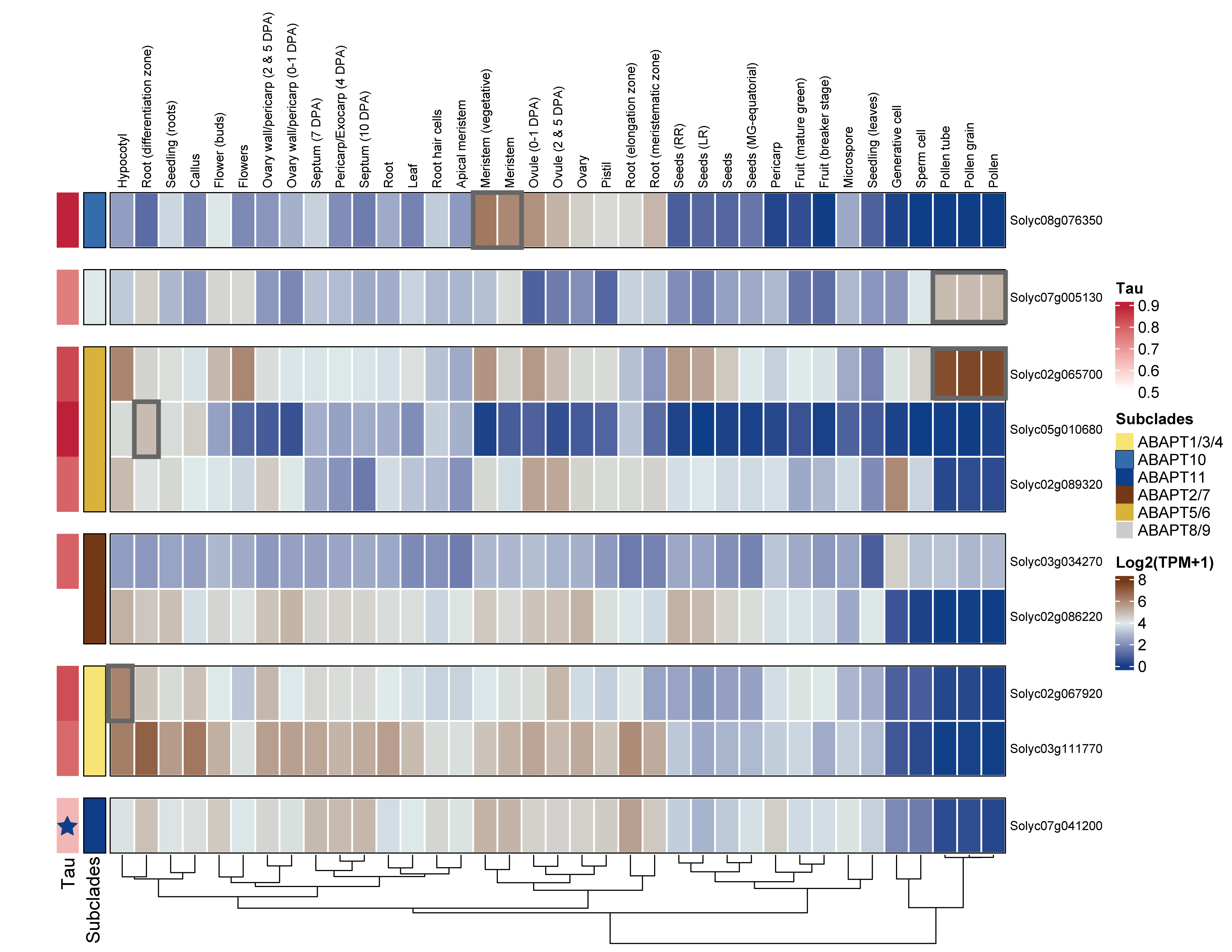

Supplement: Supplementary file 1 [file ijms-27-03691-s001.zip › Supplement Figures/Figure S13.tif]

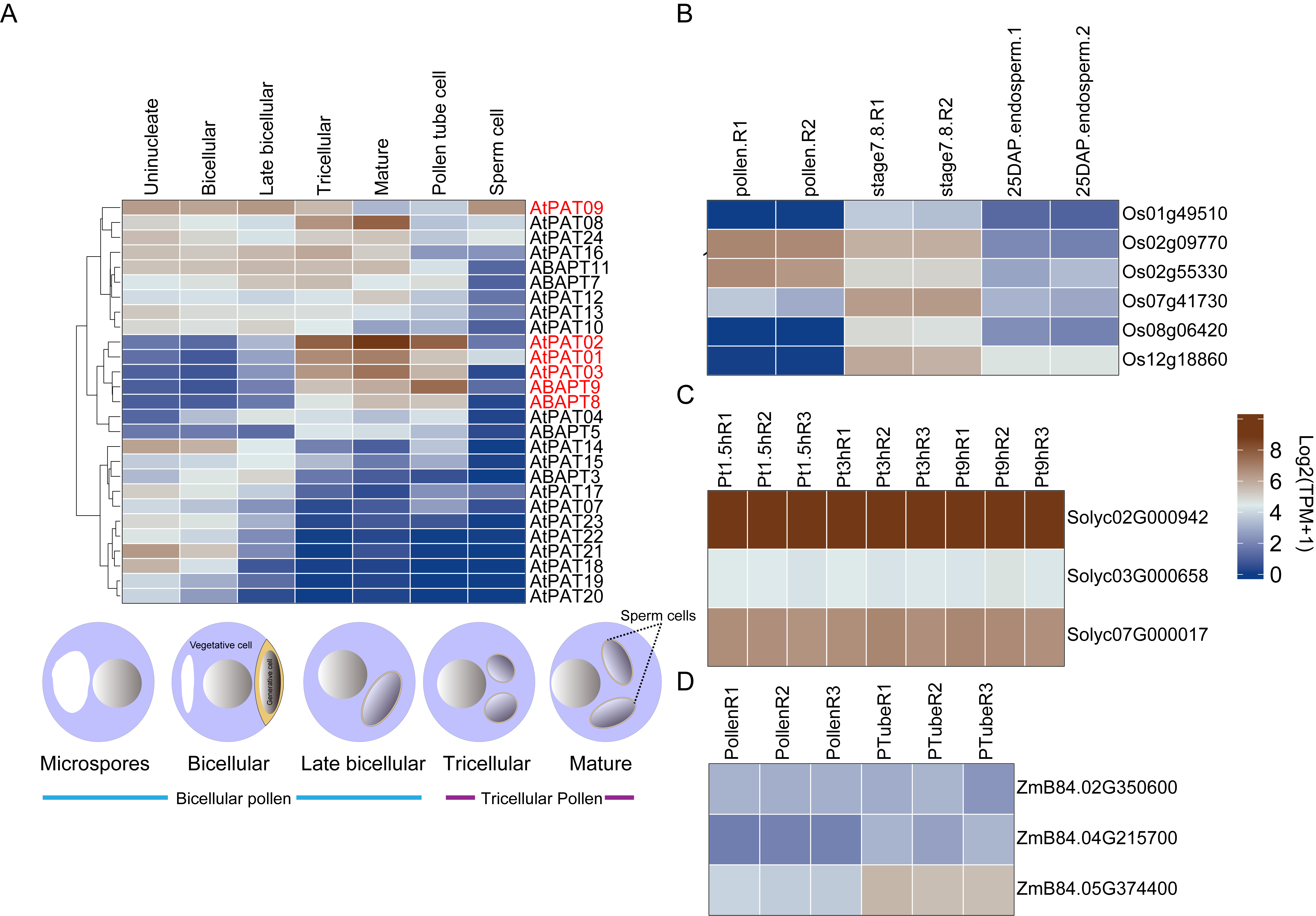

Supplement: Supplementary file 1 [file ijms-27-03691-s001.zip › Supplement Figures/Figure S14.tif]

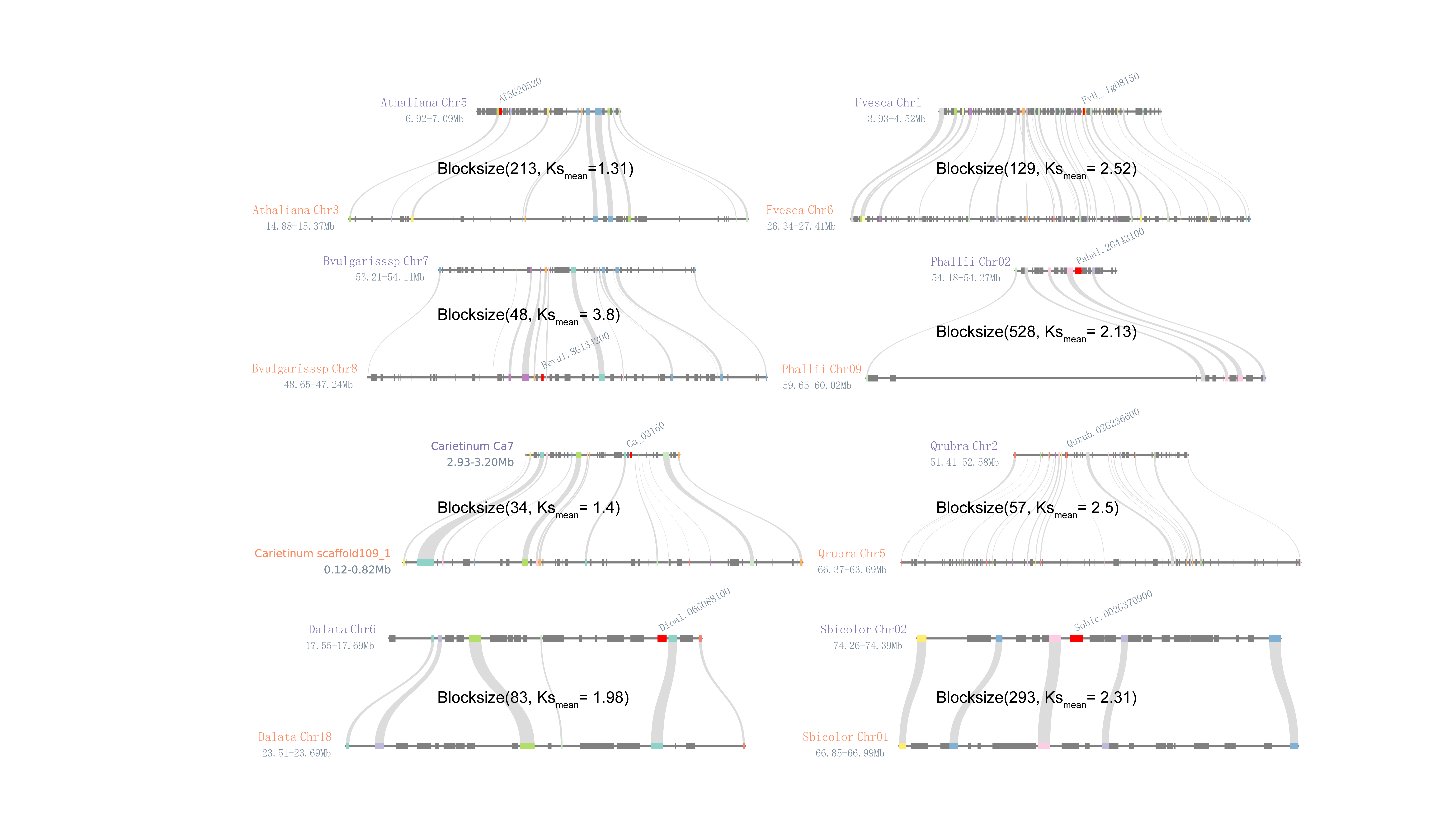

Supplement: Supplementary file 1 [file ijms-27-03691-s001.zip › Supplement Figures/Figure S2.tif]

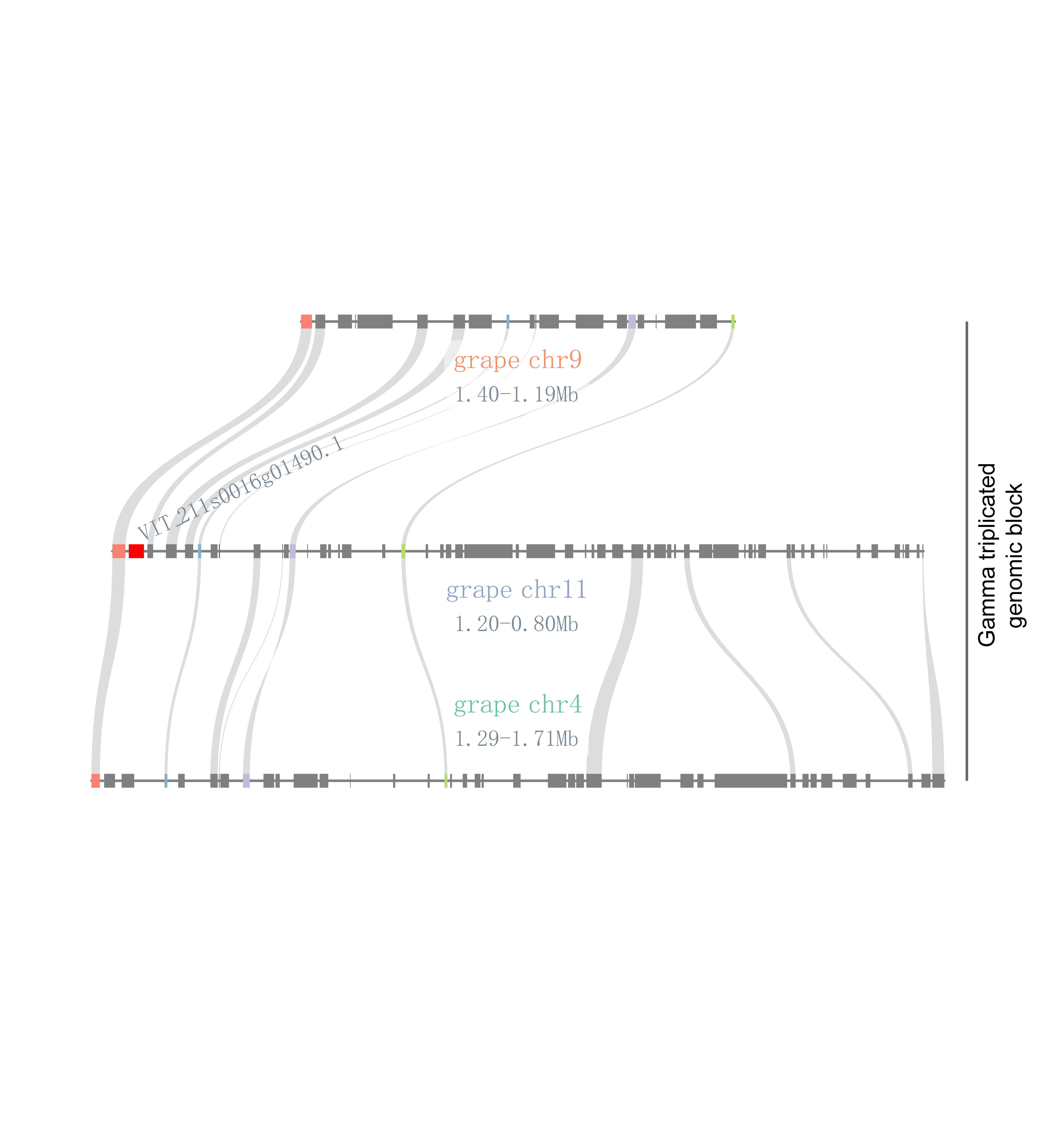

Supplement: Supplementary file 1 [file ijms-27-03691-s001.zip › Supplement Figures/Figure S3.tif]

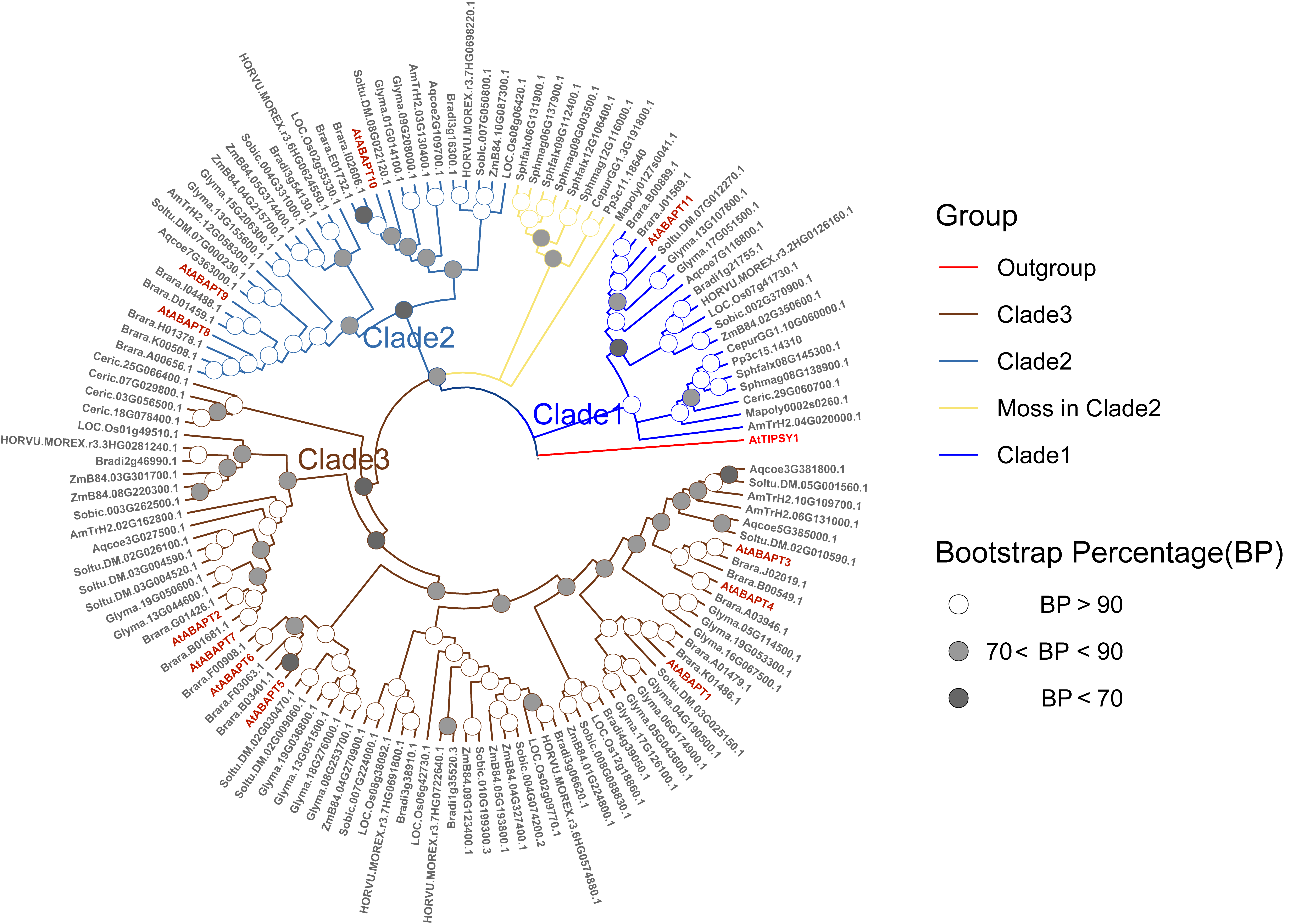

Supplement: Supplementary file 1 [file ijms-27-03691-s001.zip › Supplement Figures/Figure S4.tif]

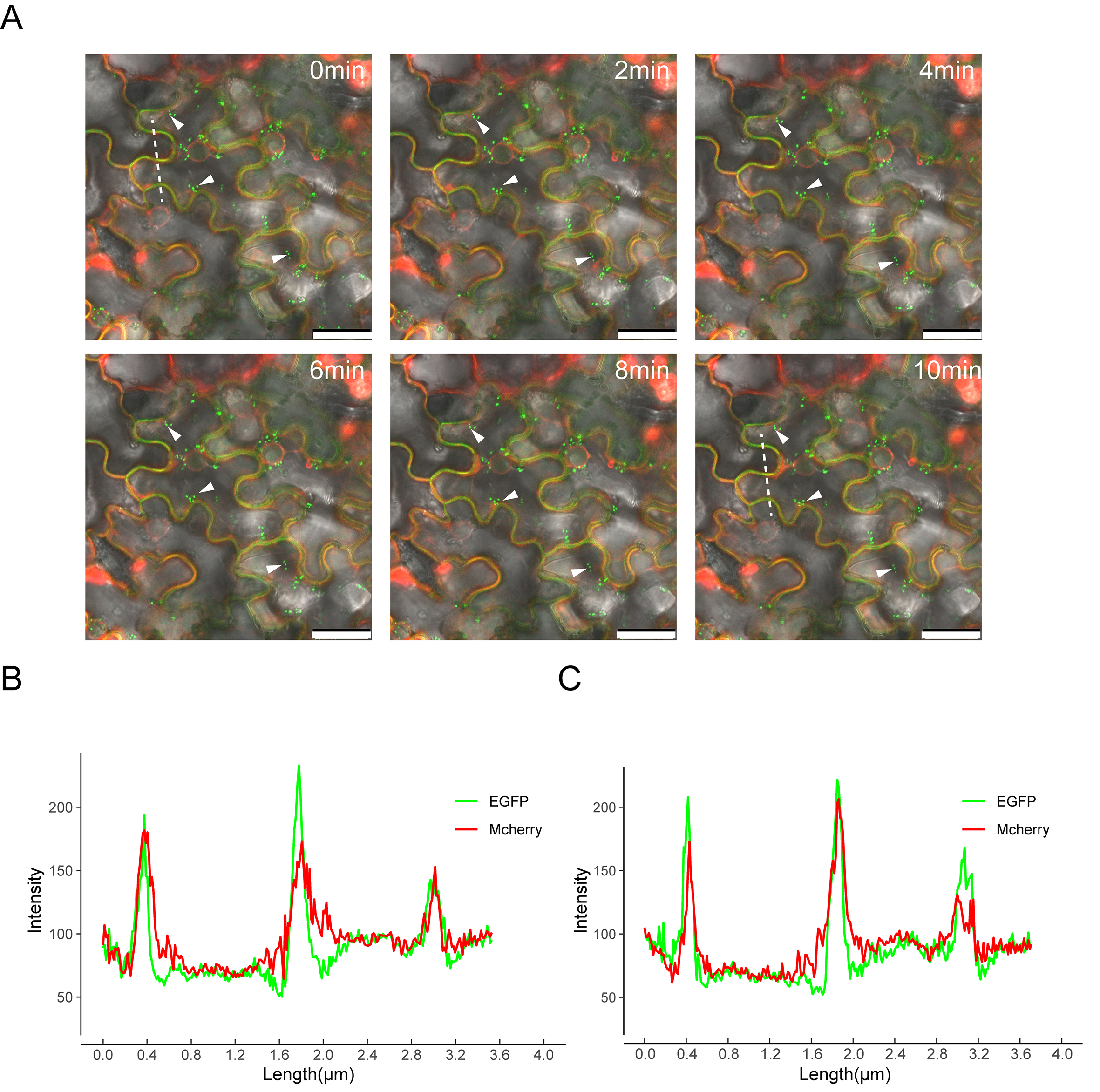

Supplement: Supplementary file 1 [file ijms-27-03691-s001.zip › Supplement Figures/Figure S5.tif]

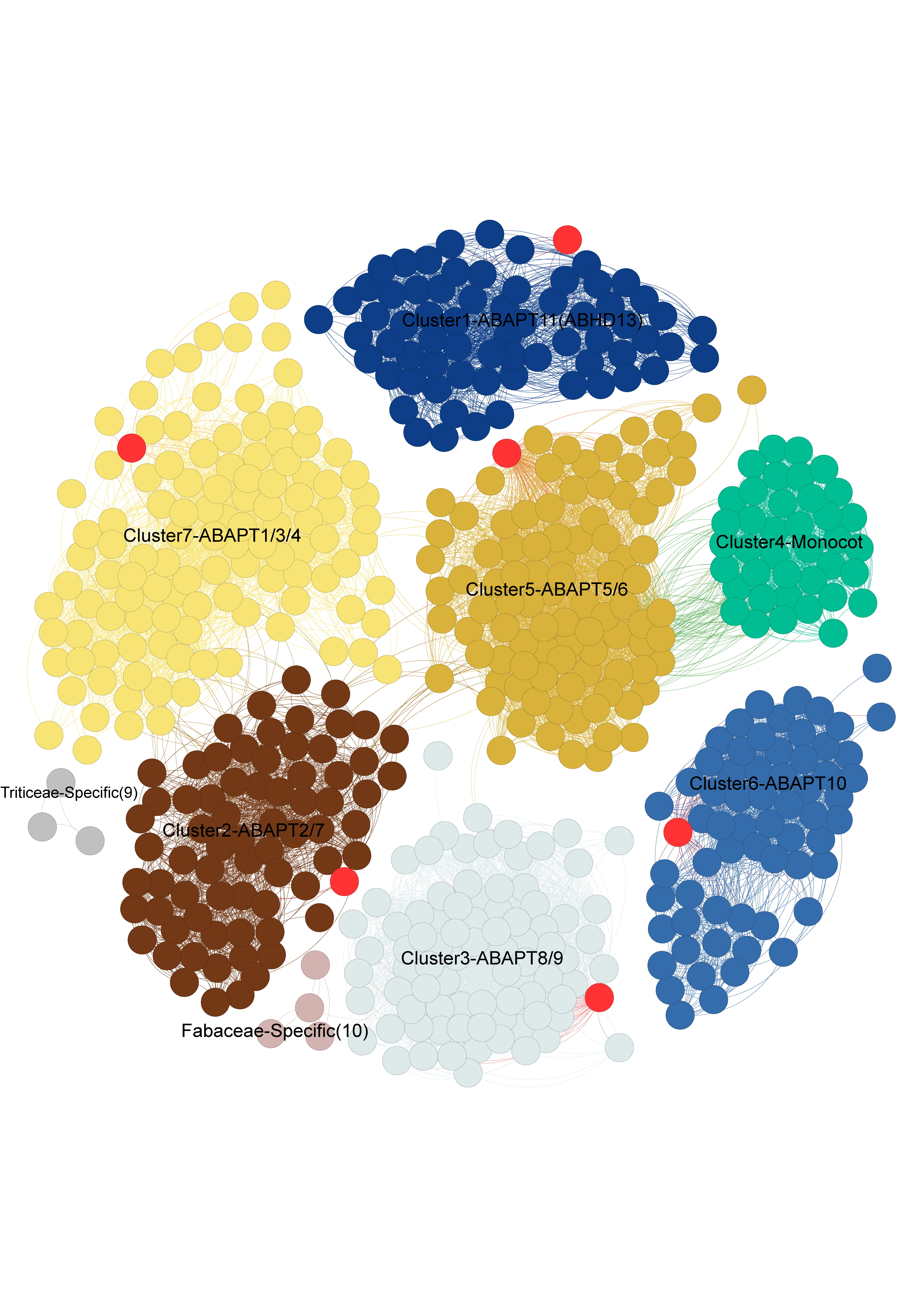

Supplement: Supplementary file 1 [file ijms-27-03691-s001.zip › Supplement Figures/Figure S6.tif]

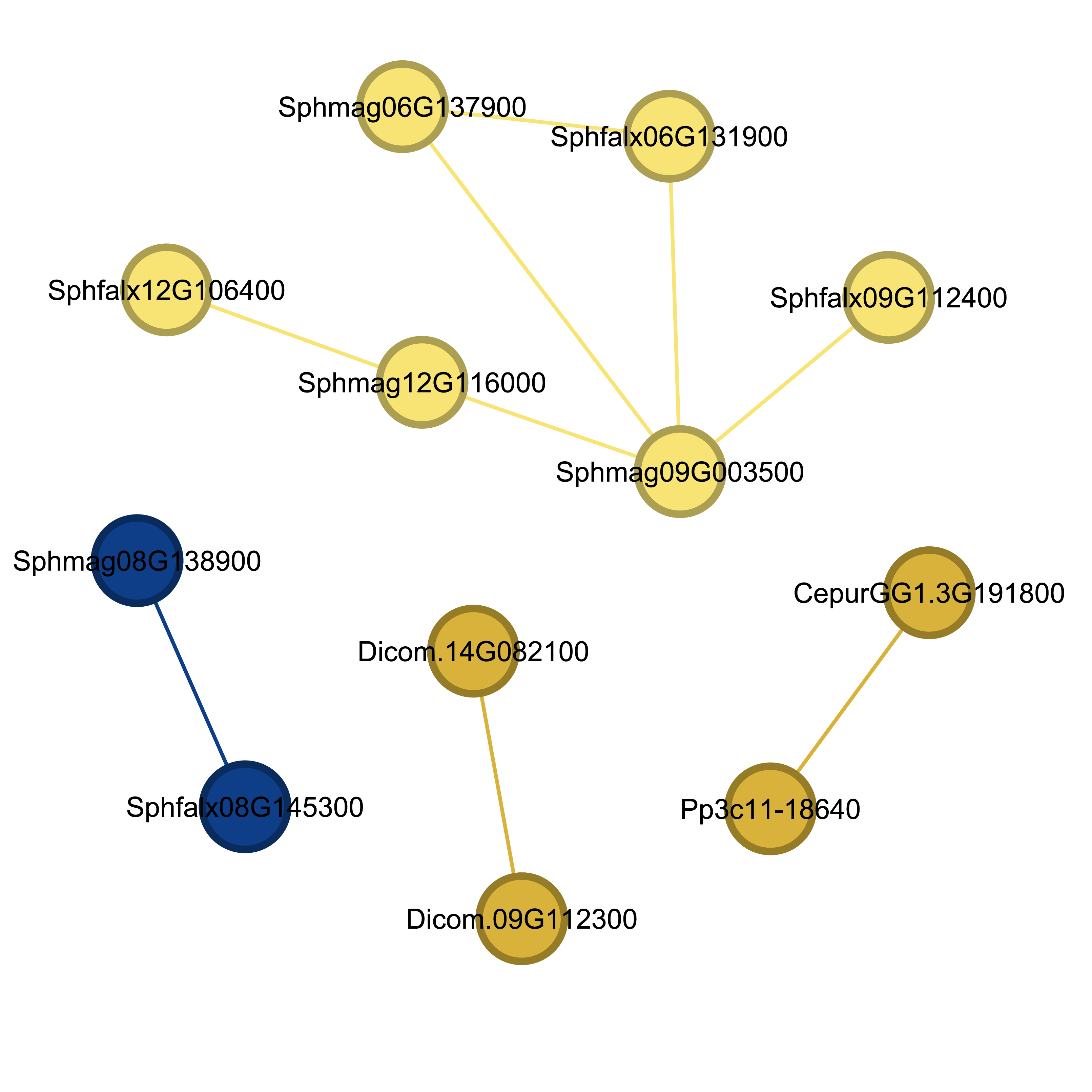

Supplement: Supplementary file 1 [file ijms-27-03691-s001.zip › Supplement Figures/Figure S7.tif]

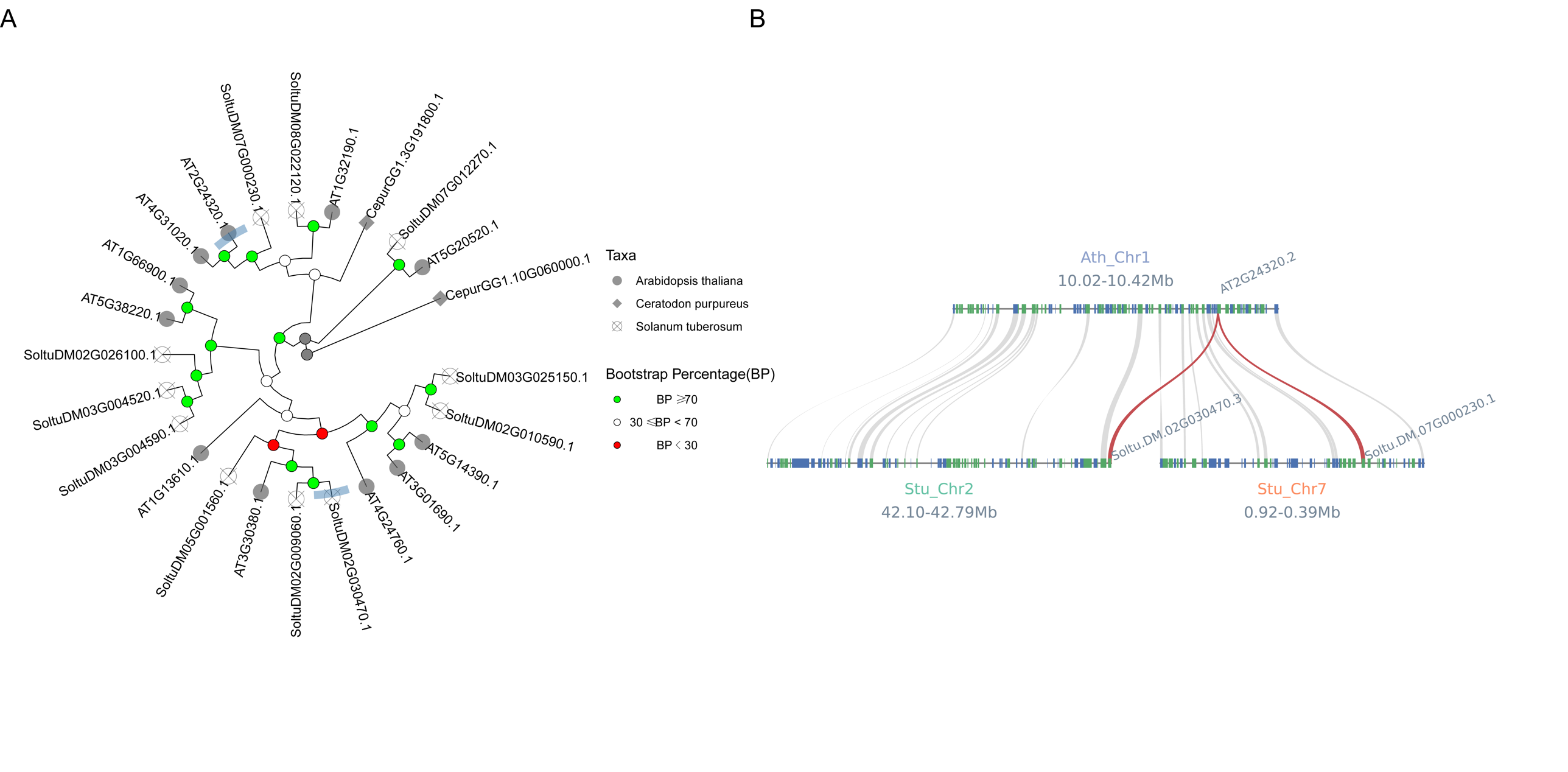

Supplement: Supplementary file 1 [file ijms-27-03691-s001.zip › Supplement Figures/Figure S8.tif]
